# Calcium Shock Enables Efficient and Programmable Particle Delivery for Genome Editing Applications

**DOI:** 10.1002/advs.202510441

**Published:** 2026-03-17

**Authors:** Nicole Vo, Lorena de Oñate, Maximillian Frank, Xian Hu, Sophie M. Blackburn, Dana V. Foss, Jasmine Villegas, Kunica Asija, Dirk Hockemeyer, Hongxia Fu, Raymond J. Monnat, Ross C. Wilson, Benjamin S. Freedman

**Affiliations:** ^1^ Division of Nephrology University of Washington School of Medicine Seattle Washington USA; ^2^ Kidney Research Institute University of Washington School of Medicine Seattle Washington USA; ^3^ Department of Medicine University of Washington School of Medicine Seattle Washington USA; ^4^ Innovative Genomics Institute University of California Berkeley Berkeley California USA; ^5^ Institute for Stem Cell and Regenerative Medicine University of Washington School of Medicine Seattle Washington USA; ^6^ CanCell Institute of Clinical Medicine Faculty of Medisin University of Oslo Oslo Norway; ^7^ Institute of Biosciences Faculty of Mathematics and Natural Sciences University of Oslo Oslo Norway; ^8^ Department of Bioengineering University of Washington School of Medicine Seattle Washington USA; ^9^ Department of Molecular and Cell Biology University of California Berkeley Berkeley California USA; ^10^ Department of Genome Sciences University of Washington School of Medicine Seattle Washington USA; ^11^ Department of Laboratory Medicine & Pathology University of Washington School of Medicine Seattle Washington USA; ^12^ California Institute for Quantitative Biosciences at University of California Berkeley Berkeley California USA

**Keywords:** amphiphilic peptides, cell‐cell junctions, CRISPR, human organoids, RNP

## Abstract

Classical intracellular delivery methods such as transfection and transduction are inefficient, particularly with confluent cells and organoids, and lack cell type‐specific programmability. We demonstrate that an innovative methodology called calcium shock (CaSh) dramatically improves particle delivery into single cells, colonies, and organoids, and enables programmable delivery (CaSh‐Pro) into specific cell types within heterocellular populations. Calcium shock works by increasing endocytotic uptake while simultaneously disarming cell‐cell junctions. CaSh‐Pro further incorporates specific molecular targeting agents and amphiphilic peptides for preferential editing of different cell types. Calcium shock improves expression of plasmid, ribonucleoprotein, or adeno‐associated viral vectors with minimal toxicity in intact organoids representing diverse lineages. CaSh and CaSh‐Pro provide simple, versatile protocols for genome editing in complex systems, to enable biological discovery and therapeutic development.

## Graphical abstract

Classical transfection and transduction are inefficient, particularly with confluent cells and organoids, and lack cell type‐specific programmability. This study presents calcium shock (CaSh), a method that dramatically improves particle delivery into single cells, colonies, and organoids. CaSh is further utilized to enable programmable delivery (CaSh‐Pro) into specific cell types within heterocellular populations.

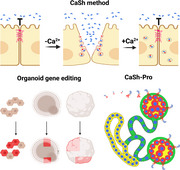

## 1 Introduction

Intracellular delivery methods such as transfection and transduction are critical tools for biological investigation that enable the introduction of large particles into cells, and are commonly used in monolayer cell cultures to achieve exogenous overexpression or conduct genome editing with CRISPR‐Cas9 [1, 2]. Such techniques have been valuable for understanding the functional ramifications of mutations, and also provide potential avenues for therapeutic delivery [3, 4]. However, the efficiency of delivery across cell membranes remains relatively low for non‐transformed cell types, e.g., human induced pluripotent stem (iPS) cells where only ∼ 5 % of cells typically take up plasmid DNA [5, 6]. An additional challenge and major unmet need is for efficient intracellular delivery protocols for intact multicellular structures such as tissues or organoids. Thus transfection, transduction, and related gene delivery methods are typically performed immediately after dissociation and replating.

In organoids that can be readily passaged, such as those containing adult stem cells, the typical approach is to dissociate such cultures prior to gene transfer, e.g., by lentiviral transduction [7]. However, this approach cannot be applied to the many organoid types that cannot be passaged. For instance, kidney organoids derived from induced pluripotent stem (iPS) cells are unable to re‐form after passaging to generate new organoids [8]. In pre‐formed kidney organoids, an adeno‐associated virus (AAV) expressing the synthetic capsid protein Anc80 has been shown to transduce mesenchymal stromal cells but not epithelia, suggesting a barrier to cell type‐specific transduction of such structures [9]. Electroporation has been utilized to introduce oncogenes into 3D aggregates containing neural stem and progenitor cells, but this has not been demonstrated to work in mature organoids [10]. Genome editing, an important method for both mechanistic investigation and therapeutic development, is particularly challenging to perform in intact organoids, due to the large size of the editing enzymes, the impenetrability of 3D structures, and the relatively low efficiency of mutagenesis even after successful delivery into cells [1, 2, 11, 12, 13, 14].

Current intracellular delivery protocols, in addition to inefficiency, also lack cell type specificity, making it difficult to target delivery to specific cell types or states, which limits both research and clinical applications [9, 13]. Organoids and tissues, in addition to being multicellular, are also heterocellular, featuring different cell types in patterned arrangements. These varied cell types can show exquisite tropisms, for example, during infection with SARS‐CoV‐2 infection that preferentially targets proximal tubular cells amongst the >10 distinct cell types in human kidney organoids [15, 16]. Distinct cell types can also manifest specific forms of disease, such as glomerulosclerosis in podocytes, or polycystic kidney disease in tubular epithelial cells [8]. More efficient, cell‐type‐specific delivery protocols would thus facilitate the investigation of cell‐type specificity of disease and potential treatments. We have previously shown that tethering of Cas9 to asialoglycoprotein receptor ligands can enhance uptake of CRISPR‐Cas9 ribonucleoprotein (RNP) complex into cells expressing the cognate receptor, providing a starting point for cell type‐specific delivery [13]. However, this approach has not yet been demonstrated for heterocellular cultures such as organoids. We hypothesized that lowering calcium levels might disrupt cellular junctions and thereby improve delivery, including the potential for programmable delivery into specific cell types [17, 18, 19].

## 2 Results

### 2.1 Calcium Shock Increases Transfection and Genome Editing in iPS Cells

**FIGURE 1:**
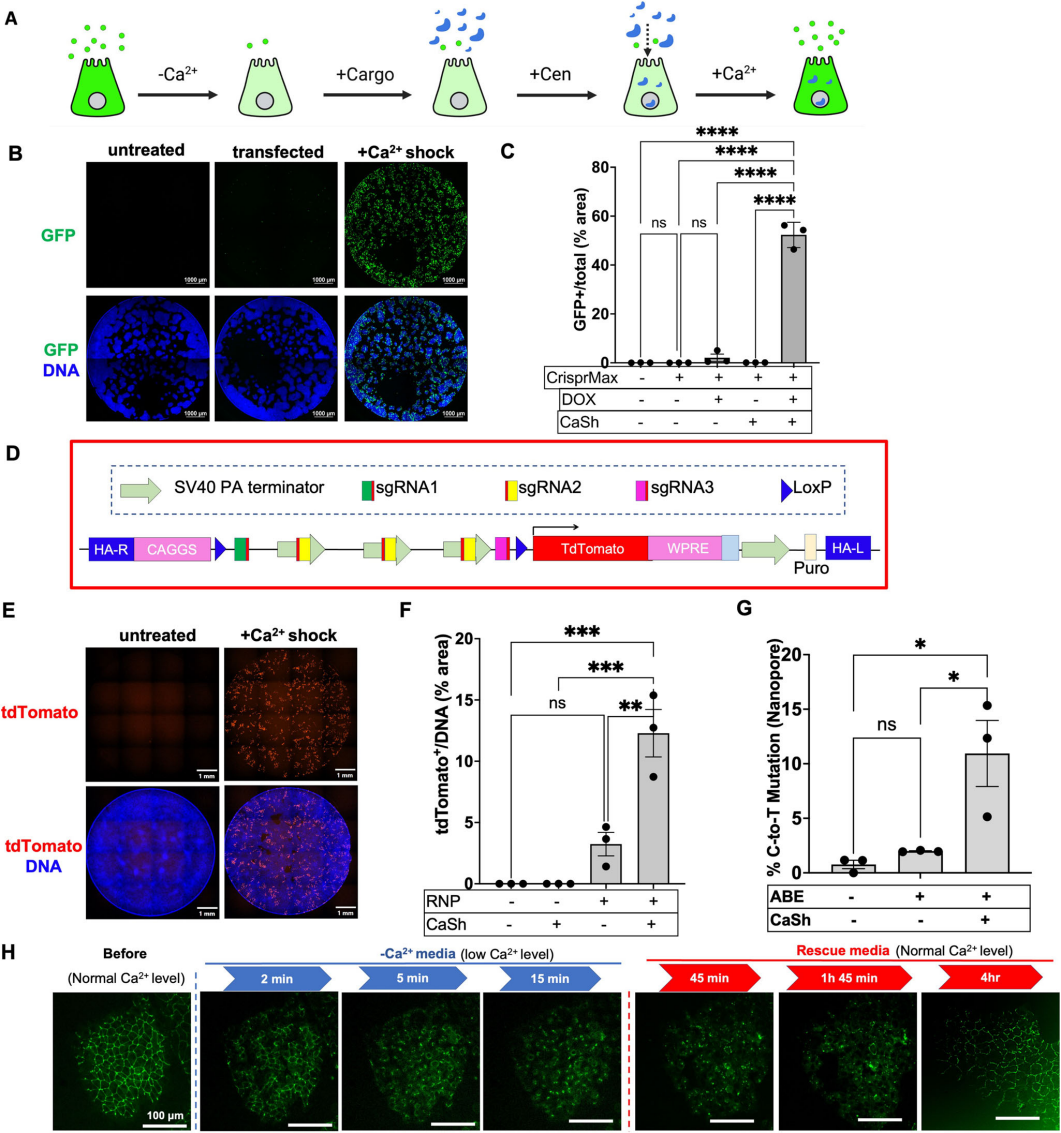
Calcium shock (CaSh) increases transfection and genome editing in iPS cells. (A) Schematic overview of calcium shock methodology. Cen, centrifugation. (B) Representative confocal immunofluorescence images of whole wells of iPS cells 72 h after transfection with a plasmid encoding doxycycline‐inducible GFP, induced with doxycycline 24 h post‐transfection, with (C) corresponding quantification (mean ± s.e.m., n = 3 independent experiments; *ns*, not significant, ^****^, *p* < 0.0001, Ordinary one‐way ANOVA with multiple comparisons). (D) Diagram of *AAVS1^LSL‐tdTom^* stop cassette construct used to generate fluorescence‐on edited iPS cells. (E) Representative confocal immunofluorescence images of whole wells of fluorescence‐on iPS cells subjected to genome editing and (F) corresponding quantification (mean ± s.e.m., n = 3 independent experiments; *ns*, not significant, ^**^, *p* < 0.01; ^***^, *p* < 0.001, Ordinary one‐way ANOVA with multiple comparisons). (G) Rates of base editing of *PKD2* in undifferentiated iPS cells, as detected using Oxford nanopore (mean ± s.e.m., n = 3 independent experiments; *ns*, not significant ^*^, *p* < 0.01, Ordinary one‐way ANOVA with multiple comparisons). (H) Representative confocal images of GFP‐expressing iPS cell colonies introduced into media without calcium for 15 min and then returned to normal calcium media.

We developed a method called calcium shock (CaSh) to enhance molecular cargo delivery into cells. Calcium shock temporarily reduces extracellular calcium levels during the particle delivery step, followed by brief centrifugation (spinoculation) and subsequent restoration of extracellular calcium levels to ∼75 % normal levels prior to overnight incubation (Figure 1A). The calcium switch is designed to temporarily dissolve cell‐cell junctions and potentially increase endocytosis. The centrifugation step is meant to increase physical contact between cargoes, which are delivered into the media, and the cells, which are adhered at the bottom of the wells. In dissociated iPS cells one day after replating, transfection of a plasmid encoding GFP in combination with calcium shock produced transgene expression in 52.3 % ± 2.1 % (mean ± s.e.m.) of cells, compared to 2.1 % ± 1.3 % (mean ± s.e.m.) without calcium shock under the same conditions (Figure 1B,C).

We next determined whether calcium shock could improve the low rates of genome editing in iPS cells [5, 6]. To measure editing more efficiently, we generated ′fluorescence‐on″ gene editing reporter iPS cells. First, we designed (a) a homologous recombination cassette encoding a Lox‐Stop‐Lox sequence preceding tdTomato at the *AAVS1* locus, and (b) a set of gRNAs targeting this Lox‐Stop‐Lox sequence for disruption (Figure 1D) [20]. Second, we integrated this cassette into the *AAVS1* locus of human iPS cells to generate the reporter (*AAVS1^LSL‐tdTom^*).

When *AAVS1^LSL‐tdTom^* iPS cells were subjected to Cas9 ribonucleoprotein (RNP) lipid‐based transfection using our gRNAs targeting the Lox‐Stop‐Lox sequence, calcium shock improved genome editing efficiency to 12.3 % ± 1.5 % (mean ± s.e.m.), a 4‐fold increase (Figure 1E,F). A similar increase in editing rate was also detected by Nanopore sequencing, using calcium shock in combination with CRISPR base editing (Figure 1G).

We hypothesized that calcium shock might improve intracellular delivery by promoting endocytosis. To visualize these effects, we used colonies of iPS cells expressing GFP‐tagged *zonula occludens*‐1 (ZO1‐GFP+), a peripheral membrane protein that localizes to tight junctions [21]. Calcium deprivation for 15 min induced a dynamic re‐localization of ZO1‐GFP from cobblestone‐like junctions into endosomal compartments, coupled with physical contraction of the cells, effects that were reversed upon calcium restoration (Figure 1H). Two transmembrane tight junction proteins, junctional adhesion molecule‐A (JAM‐A) and occludin, similarly relocalized from the plasma membrane into the same compartment as ZO1‐GFP, consistent with an endocytotic mechanism (Figure S1A,B).

Calcium shock media included basic fibroblast growth factor (FGF2), a known growth factor of human iPS cells, but FGF2 was neither necessary nor sufficient to produce the effects of calcium shock (Figure S1C). Calcium shock visibly lowered intracellular calcium levels during the transfection step (Figure S1D). Compared to standard transfection, calcium shock produced more specific intracellular and membrane‐associated localization patterns for fluorescent gRNA (ATTO550) and GFP‐tagged Cas9, respectively (Figure S1E). These data indicated that lowering the external calcium concentration promoted the uptake and internalization of transfected components, resulting in higher rates of gene expression and editing.

Some data in the literature suggest that lowering calcium may be inhibitory to endocytosis [22]. To further test the effect of calcium lowering on endocytosis, we utilized dextran–far red as a fluorescent endocytic tracer and EEA1–GFP as a marker for early endosomes in MDCK cells, a well‐established system [23, 24]. Our results showed a significant increase in dextran uptake in CaSh‐treated cells compared to a control (no CaSh), along with enhanced colocalization with EEA1–positive early endosomes (Figure S1F,G). These findings support the idea that CaSh facilitates endocytic uptake and early endosome formation.

### 2.2 Calcium Shock Increases Genome Editing in Multicellular Colonies and Organoids

**FIGURE 2:**
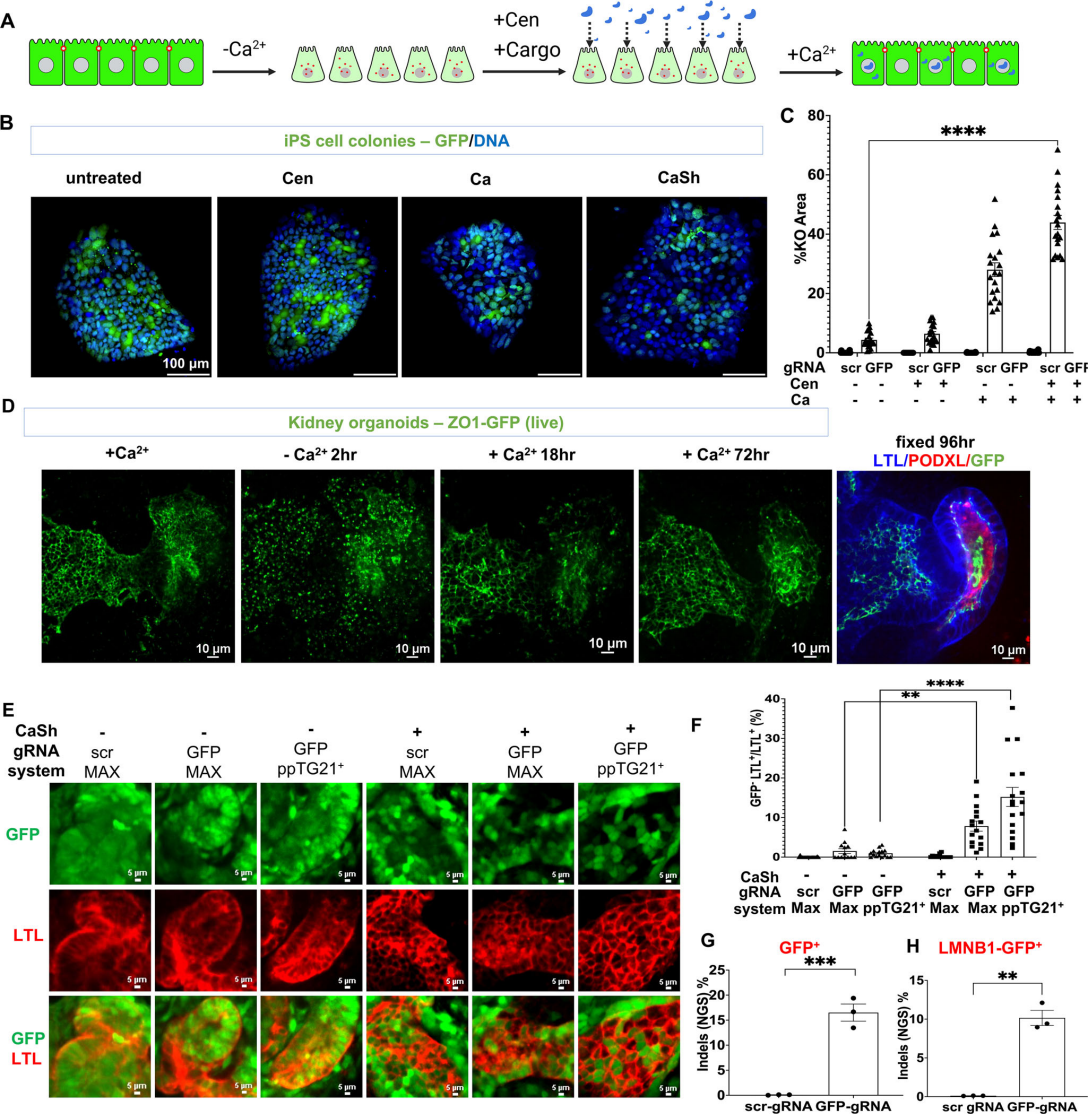
Calcium shock increases genome editing in multicellular colonies and organoids. (A) Schematic of calcium shock methodology in multicellular structures. (B) Representative confocal images of GFP‐expressing iPS cell colonies treated with RNP Cas9 gRNA with peptide ppTG21^+^ in different conditions: normal, centrifugation, calcium switch, and calcium switch followed by centrifugation (CaSh method). (C) Quantification of the percentage of knockout cells (GFP negative) in (B), compared to scrambled (scr) gRNA control (mean ± s.e.m, n = 20 images pooled from three independent experiments; ^****^, *p* < 0.0001, two‐way ANOVA with multiple comparisons). (D) Representative confocal images showing live time course and fixed immunofluorescence of representative kidney organoids expressing ZO1‐GFP subjected to calcium switch treatment (starting on day 18 of differentiation). (E) Representative confocal images of kidney organoids expressing constitutive GFP, subjected to genome editing treatments. (F) Quantification of GFP knockout cells based on immunofluorescence analysis (mean ± s.e.m., n = 17 organoids per condition, pooled from three independent biological experiments, ^**^, *p* < 0.01, ^****^, *p* < 0.0001, two‐way ANOVA with multiple comparisons). (G) Quantification of indel formation based on next‐generation sequencing (NGS) in GFP^+^ and (H) LMNB1‐GFP^+^ organoid transfection experiments (mean ± s.e.m., n = 3 independent biological experiments, ^**^, *p* < 0.01, ^***^, *p* < 0.001, Mann‐Whitney test).

Based on these effects, we investigated whether calcium shock could increase editing rates in multicellular colonies and organoids, where tight junctions pose a diffusion barrier to exposures (Figure 2A). To rapidly evaluate genome editing outcomes, we used iPS cells constitutively expressing a transgene encoding green fluorescent protein (GFP^+^) [21]. When transfected with a gene editing system targeting GFP, calcium shock significantly increased the rate of GFP loss in intact colonies as a function of calcium lowering and centrifugation (Figure S2A–C).

To further test calcium shock with a broader set of transfection reagents, we substituted CRISPRMAX (our standard transfectant) with a variety of amphiphilic peptides including E5TAT, INF7TAT‐G1K as well as peptides ppTG21, and ppTG21^+^, which belong to two families of reagents previously demonstrated to facilitate CRISPR RNP delivery, the former pair being engineered hemagglutinin‐TAT peptide fusions and the latter pair being histidine‐ and leucine‐rich peptides [13, 25, 26, 27, 28]. When used in combination with calcium shock, ppTG21 and ppTG21^+^ produced more substantial GFP knockout in colonies, compared to peptides E5TAT and INF7TAT‐G1K, or when compared to the commercially available reagent CRISPRMAX (Figure S2D). Both the calcium switch and centrifugation contributed to high efficiency of transfection with this peptide using the calcium shock protocol (Figure 2B,C; Figure S2E,F). When quantified, peptide ppTG21^+^ resulted in an impressive 45% ± 3.8% (mean ± s.e.m.) editing efficiency in intact colonies (Figure S2D–F).

To test whether calcium shock might similarly enable gene editing in intact organoids, we differentiated kidney organoids that expressed ZO1‐GFP at cell‐cell junctions of tubular epithelial cells and podocytes [8], and adjusted the timing of the calcium switch step to accommodate these larger and more complex multicellular structures. Incubation in calcium‐deficient media for 2 h induced internalization of ZO1‐GFP into endosomal foci, which could be reversed by calcium addition (Figure 2D). When GFP^+^ kidney organoids were transfected with a genome editor targeting GFP, knockout efficiency in proximal tubules was 0%–2% without calcium shock, compared to 10% or 15% when calcium shock was used in combination with CRISPRMAX or peptide ppTG21^+^, respectively (Figure 2E,F). This matched an indel formation rate of ∼16% detected by next‐generation sequencing (Figure 2G). Similar results were obtained in a second cell line expressing GFP‐tagged lamin B1 (Figure 2H) [21].

Uptake of fluorescence‐conjugated Cas9 and gRNA in kidney organoid tubules was increased to ∼70 % by calcium shock, compared to ∼25 % using standard conditions (Figure S3A–C). In contrast to calcium shock, electroporation of GFP^+^ kidney organoids with Cas9 complexes targeting GFP failed to achieve detectable gene editing (Figure S3D).

### 2.3 CaSh‐Pro Enables Programmable Delivery into Specific Cell Types in Organoids

In the preceding experiments, delivery of genome editing agents was performed in an untargeted manner, which would produce edits in many different cell types. Gene editing could conceivably be made more efficient, specific, and safe by targeting delivery of editing agents into specific cell types. Coupling of Cas9 to molecular targeting agents (MTAs) can enhance general rates of genome editing in cultured cells expressing receptors to those MTAs [13, 29]. However, targeted delivery to specific cell types has not yet been clearly demonstrated in structures with multiple cell types, such as organoids. The calcium shock fluorescence‐on system presented an opportunity to test this by targeting different compartments within kidney organoids for gene editing.

**FIGURE 3:**
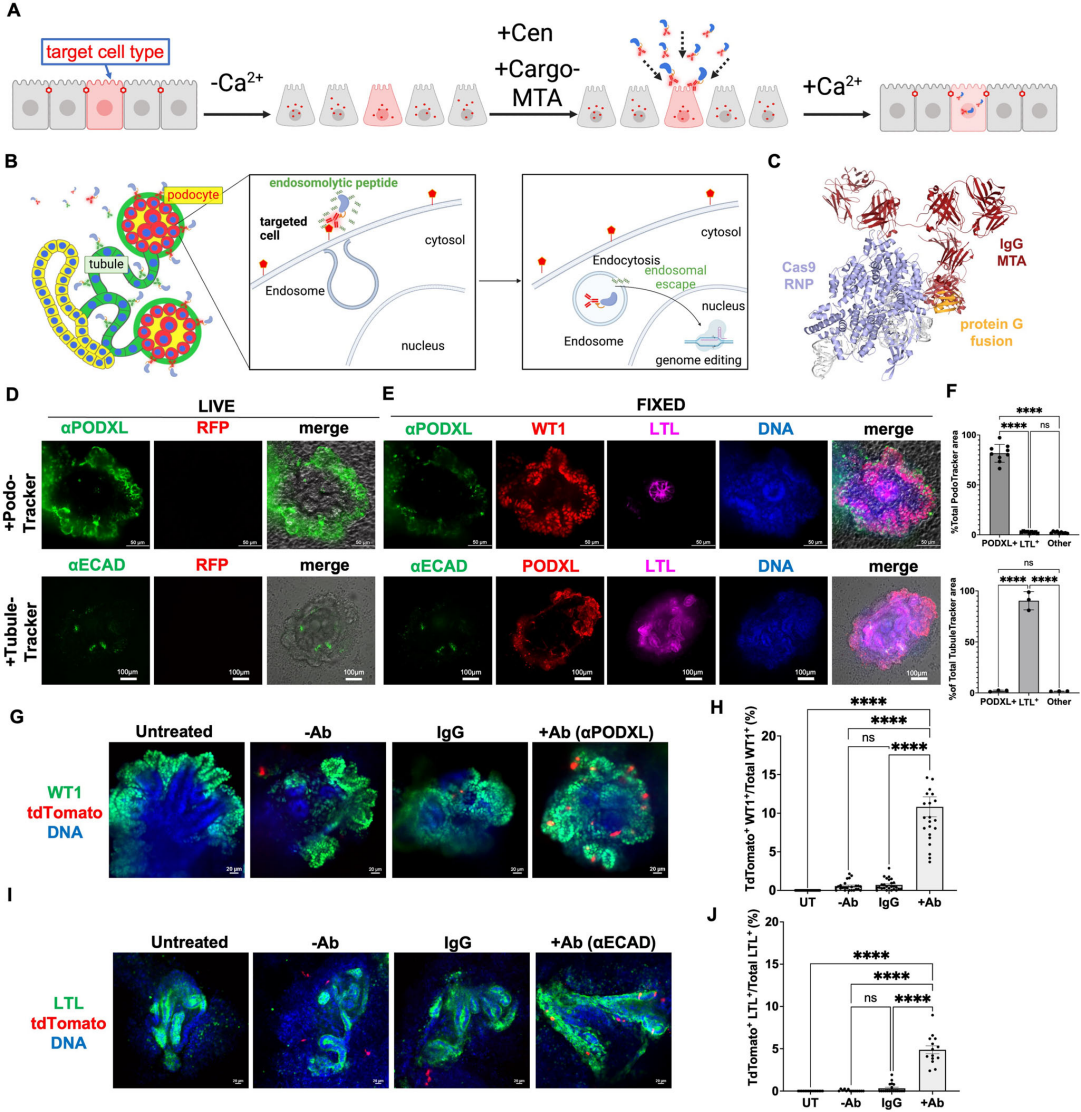
CaSh‐Pro enables programmable delivery into specific cell types in human organoids. (A) General schematic of CaSh‐Pro approach in heterocellular epithelia. (B) Detailed illustration of CaSh‐Pro mechanism in the kidney organoid structure. Cas9 complexes tethered to antibodies bind to specific cell types and are endocytosed, enabling genome editing in organoids. (C) Molecular illustration of Cas9 enzyme complex used as the basis for CaSh‐Pro. Cas9 RNP in blue/white, fused protein G domain in orange, and IgG MTA in red. The model is based on crystal structures determined for the constituent components, with respective Protein Data Bank IDs 4ZT0, 1FCC, and 1HZH. (D) Confocal live and (E) fixed fluorescence images of PodoTracker (αPODXL) or TubuleTracker (αECAD), with podocyte (WT1) and tubule (LTL) surrogate markers. (F) Quantification of the percentage of PodoTracker or TubuleTracker overlapping with PODXL^+^, LTL^+^, or other cell types (mean ± s.e.m., n=9 organoids pooled from n = 3 independent biological experiments; *ns*: not significant, ^****^, *p* < 0.0001, Ordinary one‐way ANOVA with multiple comparisons). (G) Confocal immunofluorescence images of kidney organoids under different genome editing treatment conditions, compared to untreated. Transfection of CRISPR RNP was conducted with the endosomolytic peptide E5TAT ± PodoTracker antibody. (H) Quantification of % TdTomato^+^ cells expressing the podocyte marker WT1 (mean ± s.e.m., n = 17 organoids per condition, pooled from three independent biological experiments, *ns*: not significant, ^****^, *p* < 0.0001, Ordinary one‐way ANOVA with multiple comparisons). (I,J) As for (G,H), but using TubuleTracker instead of PodoTracker, and the proximal tubule marker LTL instead of WT1 (mean ± s.e.m., n = 14–19 organoids per condition, pooled from three independent biological experiments, *ns*: not significant, ^****^, *p* < 0.0001, Ordinary one‐way ANOVA with multiple comparisons).

We took a stepwise approach to develop a version of calcium shock that was cell‐type programmable (CaSh‐Pro). First, we created a recombinant Cas9 construct fused to a fragment of protein G that tightly binds to IgG constant regions, providing a modular scaffold for targeted delivery (Figure 3A–C) [30]. Second, based on prior analyses of cell type markers in kidney organoids, we identified candidate MTAs for different kidney epithelial cell types (tubules or podocytes), labeled these with fluorophores, and then added them live to kidney organoid cultures. Third, we tested these empirically. We found that antibodies against ECAD and PODXL could serve as MTAs for tubules (TubuleTracker) and podocytes (PodoTracker), respectively, as confirmed by staining with surrogate cell type‐specific markers for these compartments after fixation (Figure 3D,E). For TubuleTracker, lotus lectin (LTL) was utilized as a surrogate marker, as these markers overlap significantly in tubular segments; for PodoTracker, Wilms tumor protein (WT1) was chosen as a surrogate marker that is enriched specifically in the nuclei of podocytes [31, 32]. Colocalization with the surrogate marker was 90.4 % ± 4.2% (mean ± s.e.m.) for and 81.6 % ± 2.5 % (mean ± s.e.m.) for TubuleTracker and PodoTracker, respectively (Figure 3F). Fourth, we further screened our repertoire of different amphiphilic peptides for their ability to produce specific live cell labeling of a fluorescence‐tagged PodoTracker tethered to Cas9‐protein G in these cultures. This screen identified an endosomolytic peptide, E5TAT, as well as CRISPRMAX, as the optimal transfectant reagents to use in this targeted delivery system (Figure S4A).

To functionally demonstrate cell type‐programmability, TubuleTracker or PodoTracker MTAs were separately tethered to Cas9‐protein G fusion ribonucleoproteins and administered to kidney organoids side‐by‐side with three negative controls: untreated cells, no included MTA, or a generic IgG instead of a specific MTA. Organoids derived from *AAVS1* ^LSL‐tdTom^ iPS cells ('fluorescence‐on') were used in these experiments as a highly sensitive and specific reporter system, in which editing events were brighter and easier to detect than in the fluorescence‐off system. The combination of E5TAT with PodoTracker or TubuleTracker MTAs resulted in substantially enhanced editing in the targeted cell types of podocytes or tubules detected, respectively, by tdTomato co‐localization with a specific marker relative to negative controls (Figure 3G–J; Figure S4B).

The editing rates in these experiments were generally lower than those observed in the non‐targeted editing experiments using CRISPRMAX described above (Figure 2F). There are two reasons for this. First, the non‐targeted editing efficiency of peptide E5TAT is lower than that of CRISPRMAX (above Figure S2D). The advantages of E5TAT were therefore limited to the context of targeted editing. Second, the detected editing rate in our fluorescence‐on system is lower than that of our fluorescence‐off system (see below). CaSh‐Pro thus enabled efficient and programmable delivery of gene editing agents into specific cell types within organoids.

### 2.4 Calcium Shock Enables Fluorescence‐On Genome Editing in Diverse Human Organoid Lineages

**FIGURE 4:**
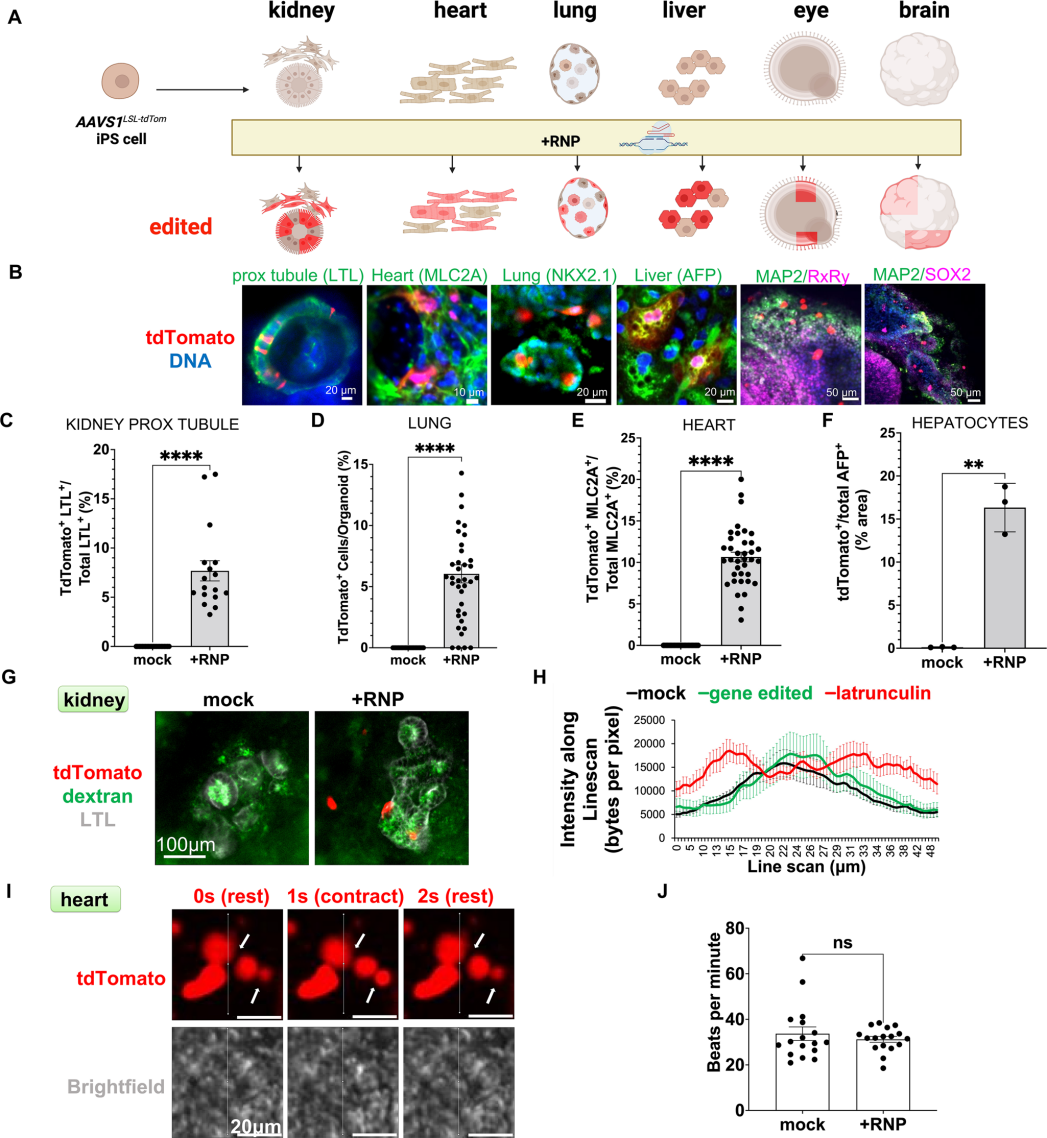
Calcium shock enables fluorescence‐on genome editing in diverse human organ lineages. (A) Schematic showing differentiation of fluorescence‐on genome editing in organ lineages. (B) Confocal immunofluorescence optical sections showing tdTomato reporter expression in different organ lineages after editing. Kidney, neuron, hepatocytes, and lung cells were transfected with peptide ppTG21^+^, and cardiomyocytes, brain, and eye cells were transfected with CRISPRMAX. (C) Quantification of tdTomato‐positive cells in proximal tubules (LTL^+^), (D) whole lung organoids, or (E) cardiomyocytes (MLC2A^+^) (mean ± s.e.m, n = 15–20 organoids per condition pooled from 3 independent experiments, ^****^, *p* < 0.0001, Mann‐Whitney test). (F) Quantification of the ratio of tdTomato positive area and AFP‐positive area in whole wells of liver cultures (mean ± s.e.m, n = 3 independent biological experiments per condition, ^**^, *p* < 0.01, Mann‐Whitney test). (G) Representative confocal immunofluorescence images of fluorescent dextran (green) in live structures overlayed with LTL staining (grey) after fixation, with (H) line scan analysis of dextran signal through kidney organoid tubules (n = 15 organoids per condition, pooled from 3 independent experiments). (I) Time‐lapse images of cardiomyocyte clusters derived from *AAVS1^LSL‐tdTom^* iPS cells transfected with Cas9 RNP and CRISPRMAX with (J) quantification of beats per minute (mean ± s.e.m. from n = 17 organoids pooled from three independent experiments; *ns*, not significant, Mann‐Whitney test).

In order to explore the generalizability of calcium shock, *AAVS1^LSL‐tdTom^* iPS cells were differentiated into a variety of lineages, including kidney, cardiomyocytes, lung, liver, eye, and brain, using established protocols and validated by qPCR, compared to undifferentiated cells as a negative control (Figure 4A; Figure S5A–D) [8, 33, 34, 35, 36]. Using calcium shock, transfection of a genome editor targeting the *AAVS1* ^LSL‐tdTom^ stop cassette resulted in focal tdTomato expression in representative cell types specific to each of these lineages (Figure 4B; Figure S5E,F) [8, 32, 37]. Rates of tdTomato induction were ∼7% in kidney proximal tubular cells and lung organoids, 11% in cardiomyocytes, and 16% in hepatocytes (Figure 4C–F). The fluorescence‐on system used in these experiments produced an editing rate in proximal tubules that was lower than that observed previously using the fluorescence‐off system (Figure 2F), but was preferred due to its high levels of specificity.

Using our tdTomato reporter, we sought to determine the broader consequences and side effects of calcium shock gene editing on organoids relating to genotoxicity, physiology, and immunological response. In kidney organoids, gene editing via calcium shock produced no detectable increase in genotoxicity or apoptosis, as assayed by immunofluorescence with gamma‐H2AX or cleaved caspase‐3, respectively, and using mitomycin C as a positive control for DNA damage (Figure S6A–D). After gene editing with calcium shock, organoids continued to accumulate dextran inside tubules, similar to untreated controls, and distinct from structures treated acutely with latrunculin (Figure 4G,H; Figure S6E) [8]. To test immunogenicity, we assessed apolipoprotein L1 (APOL1), a risk factor for kidney disease that can be triggered by inflammatory stimuli. APOL1 was not detectably increased in organoids subjected to gene editing with calcium shock (Figure S6F–G). Similarly, a sensitive qPCR assay detected no increase in the inflammatory response genes *APOL1* or *ISG15* in organoids after gene editing with calcium shock, compared to an IFNγ treated positive control, which showed 60‐fold upregulation (Figure S6H) [38, 39]. Similar to kidney organoids, cardiomyocytes expressing tdTomato after calcium shock gene editing treatments continued to beat, demonstrating the retention of key physiological hallmarks in a second organ lineage (Figure 4I,J; Figure S6I, and Movie S1). Thus, calcium shock was capable of producing gene‐edited cells without long‐term side effects.

### 2.5 Calcium Shock Increases AAV Transduction of Organoids

**FIGURE 5:**
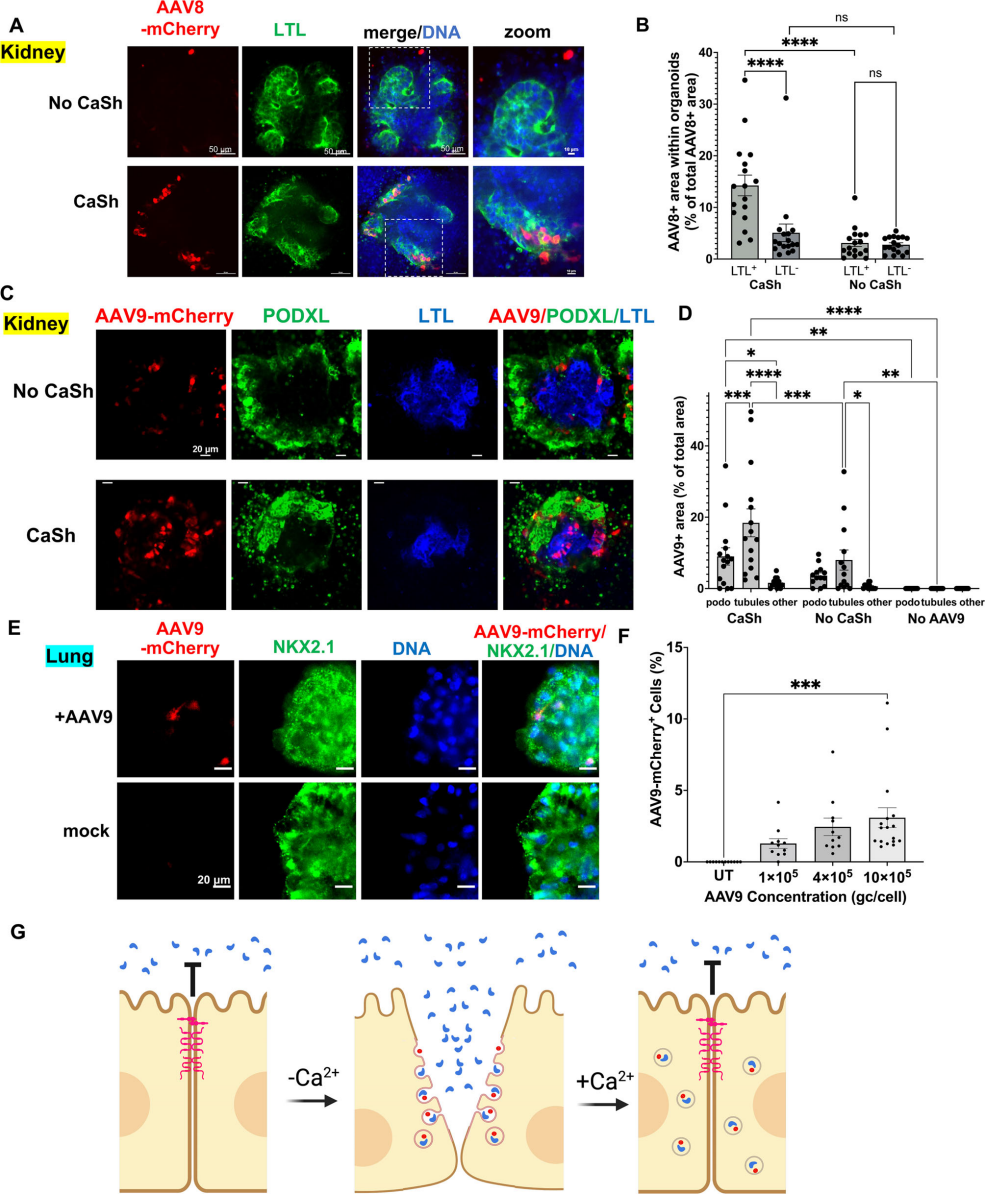
Calcium shock increases AAV transduction of organoids. (A) Representative confocal immunofluorescence of kidney organoids transduced with AAV8 ± CaSh. (B) Quantification of mCherry signal in kidney organoids transduced with AAV8 ± CaSh (mean ± s.e.m., n = 15 organoids per condition, pooled from three independent biological experiments, *ns*, not significant, ^****^, *p* < 0.0001, two‐way ANOVA with multiple comparisons). LTL+ and LTL− subpopulations are presented as separate columns. mCherry expression outside the organoids (in stromal cells) is not presented in the graph, but contributes towards the total AAV8+ area, and is thus included in the denominator but not the numerator. (C) Representative confocal immunofluorescence images of fixed kidney organoids treated with AAV9‐mCherry virus (10x10^5 gc/cell) ± CaSh. (D) Quantification of the proportion each subpopulation's (proximal tubule, podocyte, or other) area that is AAV9‐mCherry+ in kidney organoids in CaSh, No CaSh, and No AAV9 conditions (n = 15 organoids per condition pooled from 3 independent experiments, ^*^, *p* < 0.05, ^**^, *p* < 0.01, ^***^, *p* < 0.001, ^****^, *p* < 0.0001, two‐way ANOVA with multiple comparisons). Each subpopulation (x‐axis label) is segmented and quantified separately. (E) Representative confocal immunofluorescence images of AAV9‐mCherry expression in WTC11 lung organoids treated with a concentration of 10x10^5 gc/well, vs mock infection. (F) Quantification of AAV9‐mCherry+ cells in lung organoids at varying doses: 1x10^5 gc/well for low dose, 4x10^5 gc/well for medium dose, and 10x10^5 gc/well for high dose, compared to untreated control (mean ± s.e.m., n = 11–17 organoids per condition pooled from two independent experiments; ^***^, *p* < 0.001, Mann‐Whitney test). (G) Schematic model of how calcium shock promotes the delivery of cargoes into epithelial cells by temporarily dissolving cell‐cell junctions and promoting endocytotic uptake.

AAV is commonly used as a vector to transduce cells and tissues for clinical applications, but transduction with AAV is inefficient, particularly in vitro [40, 41]. In organoids, calcium shock greatly enhanced transduction of AAV8 encoding a red fluorescent reporter protein into proximal tubular epithelial cells (LTL^+^), resulting in 14.3 % ± 2.5 % (mean ± s.e.m) efficiency at 1 x 10^5^ genome copies (gc)/cell, compared to 3.1 % ± 1.3 % without calcium shock treatment (Figure 5A,B). Transduction of other cell types (LTL^−^) within the organoids was also increased with AAV8, but was not found to be statistically significant (Figure 5B). Calcium shock also enhanced transduction with AAV9, which showed high efficiency in proximal tubules (18.4 % ± 2.5 % with CaSh vs. 7.99 % ± 1.3 % without CaSh), moderate efficiency in podocytes (9.0 % ± 1.6 % with CaSh vs. 3.6 % ± 1.1 % without CaSh), and lower efficiency in other types of cells within the organoids (1.6 ± 0.5 % with CaSh vs. 0.6 % ± 0.4 % without CaSh) (Figure 5C,D). In lung organoids, calcium shock similarly increased transduction of AAV9, which showed a trend of increasing expression in proportion to dose (Figure 5E,F). These experiments emphasized the versatility of the calcium shock protocol to enhance transduction in different organoid lineages.

## 3 Conclusions

We have developed a simple protocol, calcium shock (CaSh), to improve intracellular delivery of genes and gene editing reagents into cells and organoids. Calcium shock promotes the delivery of cargoes into epithelial cells by temporarily dissolving cell‐cell junctions and promoting endocytotic uptake (Figure 5G). Interestingly, withdrawal of extracellular calcium has previously been shown to induce endocytosis of membrane junctional components [42]. Our data suggest that this response can be harnessed to improve uptake of exogenous cargoes. The calcium shock protocol is likely to be broadly useful, as it is relatively simple, inexpensive, and has been demonstrated using a variety of different cargoes, delivery methods, and organoids.

Particle delivery with calcium shock should not be confused with calcium phosphate transfection, which works by complexing DNA with calcium to form precipitates that can be taken up by cells [43]. In contrast, calcium shock enhances transfection and transduction by removing extracellular calcium to improve both access and transfer efficiency. We have not excluded the possibility that calcium precipitates may form at the stage of calcium restoration and thereby contribute to the high efficiency of calcium shock. Cells recover quickly and lack potentially deleterious DNA damage, apoptotic, functional, and immunogenic consequences. Beyond epithelia, the relationship between calcium levels and endocytosis is more complex and poorly understood. In neuronal synapses, transient, localized calcium influx appears to promote endocytosis, while prolonged, global calcium elevation can inhibit it [22].

The general mechanisms that form the basis for calcium shock can be further elaborated to allow programmable, targeted delivery into specific cell types within heterocellular organoids. The CaSh‐Pro protocol, developed to exploit this possibility, has the added advantage of being modular: Cas9 can be fused to protein G or potentially to protein A, facilitating convenient non‐covalent tethering to a wide variety of antibody MTAs. It may also be tethered to ligands that are not antibodies, as we have previously shown [13]. CaSh‐Pro may be particularly advantageous by virtue of its cell type‐specificity of delivery and potential to enhance delivery and editing while minimizing collateral damage to other adjacent cell types.

Genome editing systems such as CRISPR‐Cas9 typically produce editing rates an order of magnitude lower or more than transfection rate [2, 11, 12]. This likely reflects the challenge of trafficking large editing complexes out of endosomes and into the nucleus. Endosomal escape is typically achieved by only ∼0.01% of cargoes, which makes this step rate‐limiting for successful cytosolic delivery of complexes such as RNP [13, 14]. We have developed editing‐dependent fluorescence‐‘off’ and fluorescence‐‘on’ reporter iPS cell lines to enable optimization of both calcium shock and CaSh‐Pro in different types of organoids. Of note, editing rates observed with our fluorescence‐on reporter were lower than with fluorescence‐off. This may be due to differences in the inherent editing potential of the two distinct loci, or alternatively may indicate differences in accuracy between the two systems, the fluorescence‐on system being the more stringent of the two. Both of these reporters likely underestimate actual editing rates, as only a portion of edits will lead to detectable editing‐dependent fluorescent protein expression [2, 44]. These reporter lines have nonetheless enabled us to identify several different amphiphilic peptides that improved the efficiency of calcium shock or CaSh‐Pro, leading to editing rates of ∼10%, which could be useful in a clinical setting. This may suffice in, for example, cases of stem cell editing where successful editing of only a small fraction of cells has the potential to rescue a disease phenotype by conferring a proliferative fitness advantage or by complementing a systemic deficiency, for instance in sickle cell disease, muscular dystrophy, hemophilia A, or cystic fibrosis [45, 46, 47, 48, 49]. Calcium shock protocols may also be of great value in instances where it is necessary to edit a larger percentage of cells in order to produce a therapeutic benefit.

Of note, CaSh also enhances transduction of human kidney organoids with AAV8 and AAV9. In the absence of Cash, transduction of kidney organoids with these capsids is reported to be negligible compared to AAV2, which exhibits tropism for proximal tubules [50]. Our data also indicate that AAV8 and AAV9 exhibit tropism for proximal tubules and podocytes compared to other types of cells within the organoids, and this tropism may be enhanced by CaSh. AAV tropism is defined in part by capsid‐cell surface interactions, which are influenced by receptor availability, membrane dynamics, and endocytosis pathways. Calcium depletion, mechanical stress (via spinoculation), or altered membrane fluidity during the CaSh protocol could potentially modify cell surface receptor exposure or internalization pathways, thereby altering the apparent tropism or pseudotyping behavior of AAV vectors. This may be an interesting direction for future research.

An important future direction for calcium shock development is to fully explore how they can be used to enhance human ex vivo and in vivo gene editing, which we have not yet investigated. Genome editing of cells ex vivo prior to implantation is the technological basis for CASGEVY (exagamglogene autotemcel), the first CRISPR‐enabled therapy approved by the United States Food and Drug Administration [51, 52]. Several therapeutics based upon genome editing of chimeric antigen receptor T‐cells are also in clinical trials [53, 54]. Calcium shock approaches described herein are well positioned to aid the development of such technologies ex vivo by increasing the efficiency or selectivity of gene editing. CaSh‐Pro could be particularly useful in vivo to conduct targeted delivery of therapeutic agents (e.g., genome editors) to specific cell types, for instance, using well‐understood calcium chelating agents such as EGTA and BAPTA to transiently lower calcium levels in vivo prior to gene editing [55, 56].

Before this vision can be realized, it will be important to overcome current technical and safety challenges associated with calcium modulation in living tissues. Several aspects currently limit the feasibility of in vivo CaSh, including potential systemic and local adverse effects of transiently altering extracellular calcium levels in tissues, which could disrupt normal cellular functions, cell‐cell junctions, and homeostasis; unknowns regarding the pharmacokinetics and biodistribution of CaSh reagents and procedures; and challenges in targeting calcium shock effects to specific tissues or cell populations without off‐target consequences. Thus, it will be important to conduct comprehensive preclinical evaluations prior to attempting an experiment in people.

The resemblance of many organoids to human tissues may allow gene therapy protocols to be developed and optimized in organoids prior to such testing in vivo. The ability of calcium shock to enhance gene delivery to hard‐to‐edit cells and target specific cell types in organoids suggests this technology will be broadly enabling for both basic and translational research.

## 4 Methods

### 4.1 Experimental Models and Study Participant Details

#### 4.1.1 Ethics

All activities were approved by appropriate review boards within the respective institutions. Research complied with all relevant ethical regulations. iPS cell culture and differentiation were conducted under the approval of the University of Washington Embryonic Stem Cell Research Oversight Committee. All cell lines were generated with informed consent.

#### 4.1.2 iPS Cell Maintenance Culture

Specific cell lines used in this study are described in the manuscript and include WTC11 iPS cells expressing GFP (Allen Institute/Coriell AICS36, RRID: CVCL_JM19), LMNB1‐GFP (AICS13, endogenous lamin B fused with GFP, RRID: CVCL_IR32), ZO1‐GFP (AICS23, endogenous *zonula occludens* 1 fused with GFP, RRID: CVCL_JM18), and *AAVS1* ^LSL‐tdTom^ iPS cells (WIBR3, RRID: CVCL_9767 Hockemeyer lab, UC Berkeley). All lines were tested and found to be negative for contamination (Coriell Biorepository and Hockemeyer laboratory) [20]. iPS cell cultures were maintained in mTeSR1 (STEMCELL Technologies, 85850) media that was exchanged daily. Cells were passaged weekly using Accutase (STEMCELL Technologies, 07920) or ReLeSR (STEMCELL Technologies, 05872).

### 4.2 Method Details

#### 4.2.1 Kidney Organoid Differentiation

750–1500 iPS cells per well in mTeSR1 (STEMCELL Technologies) + 10 µm ROCK inhibitor were placed in 96‐well plates pre‐coated with 60 µL of DMEM‐F12 (Gibco, 11320‐033) containing 0.2 mg/mL GelTrex, and sandwiched the following day with 0.3 mg/mL GelTrex in mTeSR1 (STEMCELL Technologies, 85850) to produce scattered, isolated spheroid colonies. Resulting iPS cell spheroids 48 h later were treated with 12 µm CHIR99021 (Tocris Bioscience) for 36 h, then changed to RB (Advanced RPMI + 1X Glutamax + 1X B27 Supplement, Thermo Fisher Scientific) medium and refed every 2 days thereafter. Organoids were differentiated under static culture conditions for 18 days from the time of iPS cell plating prior to genome editing.

#### 4.2.2 Lung Organoid Differentiation

*AAVS1^LSL‐tdTom^* iPS cells (800 000 cells/well) were placed in low attachment 6‐well plates with embryoid body (EB) media consisting of Serum Free Differentiation (SFD) basal media (3:1 IMDM (Corning, 10‐016‐CV)/Hams F12 (Corning, 10‐080‐CV) with added 1x N2 (Gibco, 17502‐048), 1x B27 (Thermofisher, 17504044), 0.05625% BSA (Gibco, 15260037), 1x Penicillin‐Streptomycin (Thermofisher, 15‐140‐122), 1x Glutamax (Thermofisher, 35050061) and 0.4 µm monothioglycerol (Sigma Aldrich, M6145)), to which 10 µg/mL Y‐27632 (Tocris Bioscience, 1254), 3 ng/mL BMP4 (R&D Systems, 314‐BP) and 50 µg/mL ascorbic acid (Sigma Aldrich, A‐4544) were added prior to culture in a low oxygen (5% oxygen) incubator for 16 h.

Resulting EBs were transferred into Embryoid Induction Media (EIM) made of SFD basal media supplemented with 10 µg/mL Y‐27632, 0.5 ng/mL BMP4, 50 µg/mL ascorbic acid, 2.5 ng/mL β‐FGF (Peprotech, AF‐105‐18B), and 100 ng/mL Activin A (Peprotech, 120‐14P) prior to incubation in low oxygen (5% oxygen) incubator. After 24 h, 1 mL/well of EIM was added, followed 24 h later by an additional 2 mL/well of EIM. After another 48 h incubation, embryoid bodies were dissociated into single cell suspension using 0.5 mL/well of 0.5% Trypsin/EDTA (Life Technologies, 25200056) at 37^0^C for 3 min. The trypsin mixture was neutralized with 5 mL Stop Media (IMDM with 5% FBS, 1x Glutamax, and 1x Penicillin‐Streptomycin). After centrifugation at 376 rcf for 4 min, cells were plated at 0.75 x 10e6 cells/well in fibronectin (4µg/ml)(R&D Systems, 1918‐FN)‐coated 6‐well plates (Fisher Scientific, 07‐200‐83) and grown in Anteriorization media 1 (AM1: consisting of SFD basal media supplemented with 10 µm SB‐431542 (Caymen Chemical Company, 13031), 100 ng/mL Noggin (Peprotech, 120‐10C) and 50 µg/mL ascorbic acid), prior to culture in a normoxic incubator (37°C, 20% O_2_, 5% CO_2_) for 24 h. AM1 was then replaced with 2 mL/well of Anteriorization media 2 (AM2: SFD basal media supplemented with 1 µM SB‐431542, 1 µm IWP2 (Tocris, 3533), and 50 µg/mL ascorbic acid) prior to incubating for an additional 24 h. AM2 was then replaced with 2 mL/well of branching media (BM: SFD supplemented with 3 µm CHIR99021 (Tocris, 4423), 10 ng/mL BMP4, 10 ng/mL FGF10 (R&D Systems, 345‐FG), 10 ng/mL FGF7 (R&D Systems, 251‐KG), 50 nm *all‐trans* Retinoic acid (Tocris, 0695) and 50 µg/mL ascorbic acid), followed by normoxic incubation for 48 h. Colonies were harvested by replacing media with 2 mL/well of fresh BM prior to shearing colonies off substrate to grow in suspension in ultra‐low attachment 6‐well plates (Corning, 3471). The resulting organoids were then fed every other day until branching structures were formed at 18 days for the WTC11 cells and 28 days for *AAVS1^LSL‐tdTom^* cells.

### 4.3 Cardiomyocyte Differentiation

Human *AAVS1* ^LSL‐tdTom^ iPSCs were propagated on Geltrex under feeder‐free conditions in mTeSR1 (STEMCELL Technologies, 85850) + 10 µm ROCK inhibitor (BD Biosciences, 562822) at 3000–3600 cells/well in 96‐well plates (Greiner Bio‐one, 655090). Within 3 days, confluency reached 70%, and we induced differentiation by adding GSK3β inhibitor CHIR99021 (Stemgent, 04‐0004‐10) at a final concentration of 7 µm in fresh RB without insulin media (RPMI media (Gibco, 12633‐012) + 1X B‐27 without insulin (Gibco, A18965‐01) + 1X Glutamax (Gibco, 35050061). Exactly 24 h after the addition of CHIR99021, cells were fed with fresh RB media. 48 h thereafter, IWP2 (Tocris, 3533) was added at a final concentration of 5 µM in fresh RB media. Fresh RB was provided 48 h after the addition of IWP2, and once more 48 h after that. Henceforth, the cells were maintained by replacing the media with fresh RB every 3 days. Aggregates of beating cardiomyocytes were observed by day 9 after CHIR99021 addition, and by day 12 spontaneous contractions could be seen in large areas of the well.

### 4.4 Liver Differentiation

Human liver cultures were differentiated using the StemDiff Hepatocyte Differentiation Kit (STEMCELL Technologies, 100‐0520). Human *AAVS1* ^LSL‐tdTom^ iPSCs were propagated on Geltrex under feeder‐free conditions in mTeSR1 (STEMCELL Technologies, 85850) + 10 µm ROCK inhibitor (BD Biosciences, 562822) at 15 000 cells/well in 96‐well plates (Greiner Bio‐one, 655090). On day 1, a full medium change with 100 µL of Medium 1 was performed per well. From days 2–4, a full medium change with 100 µL of Medium 2 was performed per well. From days 5–9, a full medium change with 100 µL of Hepatic Progenitor Medium was performed per well. On day 10 and every other 2 days thereafter, a full medium change with 100 µL of Hepatocyte Medium was performed per well. On day 21, hepatocytes were ready for treatment with genome editors.

### 4.5 Brain and Retinal Organoid Differentiation

*AAVS1^LSL‐tdTom^* iPS cells (3 000 000 cells/well) were placed in 6‐well plates pre‐coated with 60 µL of DMEM‐F12 (Gibco, 11320‐033) containing 0.2 mg/mL GelTrex. iPS cell cultures were maintained in mTeSR1 (STEMCELL Technologies #85850) media exchanged daily until 90% confluent. Media was replaced with Essential 6 (E6, ThermoFisher #A1516401) media for 2 consecutive days. On day 3 of differentiation, E6 media was replaced with a pro‐neural induction media (PIM, composed of advanced DMEM/ F12, 1% N2 supplement, 1% L‐glutamine, 1% non‐essential amino acids, and 1% antibiotic‐antimycotic). By 4 weeks of culture, 3D organoids containing rosettes were observed throughout the plate and in close proximity to neuroretinal vesicles, and were switched into retinal differentiation media (RDM; composed of DMEM, 30% F12 nutrient mix, 2% B27‐vitamin A, and 1% antibiotic‐antimycotic). At 6 weeks of differentiation, RDM was supplemented with 10% fetal bovine serum (FBS), 100 mm taurine, and 2 mm GlutaMAX and maintained in this media until fixation. On week 7, organoid cultures were treated with genome editing agents ± calcium shock.

### 4.6 Cas9‐RNP Transfection in Human iPS Cells

Undifferentiated AICS36 iPS cells at 3 000 cells/well (for colonies) or 10 000 cells/well (for single cells) were plated in 96‐well plates pre‐coated with 60 µL of DMEM‐F12 (Gibco, 11320‐033) containing 0.2 mg/mL GelTrex. 24h (for single cells) or 48 h (for colonies) after seeding, cells were transfected with 10 pmol of Alt‐R S.p. HiFi Cas9 Nuclease V3 (IDT, 1081061), 12 pmol of sgRNA targeting GFP (aacgtctatatcatggccgacaa, IDT) or scrambled guide (Alt‐R CRISPR‐Cas9 Negative Control crRNA #1, IDT, 1072544) and either Lipofectamine CRISPRMAX Cas9 Transfection Reagent (1.5 µl of Cas9 Plus and 0.9 µl of CRISPRMAX, both from Thermo Fisher, #CMAX00008) or 100 pmol peptides ppTG21, ppTG21^+^, E5TAT, and INF7TAT‐G1K (Wilson lab) in 60 µl of calcium switch media (DMEM 1X (21068‐028, Gibco) supplemented with 1X GlutaMAX (35050061, Thermo Fisher), 1x Sodium Pyruvate 100 mm (11360‐070, Gibco), 10 ng/ml Recombinant Human FGF‐basic (100‐18B, Peprotech) and 40 µl volume of mTESR1 (STEMCELL Technologies, 85850)). Cells were incubated for 5 min and then were centrifuged in the 96‐well plate at 120 rcf and 37°C for 15 min. After centrifugation, 100 µl of Opti‐Mem media (Thermo Fisher, 1985062) was added to each well, then replaced with 200 µl of mTESR1 medium 24 h after transfection.

### 4.7 Base Editing in Human iPS Cells

Undifferentiated *AAVS1* ^LSL‐tdTom^ iPS cells at 15 000 cells/well were plated in 96‐well plates pre‐coated with 60 µL of DMEM‐F12 (Gibco, 11320‐033) containing 0.2 mg/mL GelTrex. 24 h after seeding, cells were co‐transfected with pEF‐AncBE4max (90 ng), pDT‐sgRNA with unique guide PKD2‐R872X (5’‐TGGAGCGAGCCAAACTGAAG‐3’) (30 ng), and pEF‐BFP (30 ng) using 0.5 µl Lipofectamine Stem transfection reagent (Themo Fisher, STEM00008) in a total 100 µl volume of calcium switch media (DMEM 1X (21068‐028, Gibco) supplemented with 1X GlutaMAX (35050061, Thermo Fisher), 1x Sodium Pyruvate 100 mm (11360‐070, Gibco), 10 ng/ml Recombinant Human FGF‐basic (100‐18B, Peprotech)). Cells were incubated for 5 min and then were centrifuged in the 96‐well plate at 120 rcf and 37°C for 15 min. After centrifugation, 100 µl of Opti‐Mem media (Thermo Fisher, 1985062) was added to each well, then replaced with 200 µl of mTESR1 medium 24 h after transfection.

### 4.8 Electroporation of Fluorescence‐Off Kidney Organoids

On day 14 of differentiation, 25 kidney organoids expressing GFP in a 96‐well plate were microdissected from their original plates and then electroporated with Cas9‐RNP complex (10 pmol of Alt‐R S.p. HiFi Cas9 Nuclease V3 (IDT, 1081061), 9 pmol of sgRNA targeting GFP (aacgtctatatcatggccgacaa, IDT) in 100 µl of electroporation media inside a nucleofection cuvette using the Basic Nucleofector Kit for Primary Mammalian Epithelial Cells (Lonza) according to instructions on an Amaxa device. After nucleofection, 500 µl of RB media was added immediately to the cuvette, and the entire mixture was transferred into a falcon tube containing 2.5 mL RB. 2 mL of this media containing the organoids was subsequently plated in one well of a 24‐well plate, pre‐coated with 0.2 mg/mL GelTrex (Invitrogen) [57]. 48h after re‐plating, the cells were imaged using confocal microscopy.

### 4.9 Fluorescence‐Off Editing of Intact Organoids

Kidney organoids expressing GFP or GFP fusion proteins on day 18 of differentiation in a 96‐well plate were transfected with 55 pmol of Alt‐R S.p. HiFi Cas9 Nuclease V3 (IDT, 1081061), 45 pmol of sgRNA targeting GFP (aacgtctatatcatggccgacaa, IDT) or scrambled guide (Alt‐R CRISPR‐Cas9 Negative Control crRNA #1, IDT, 1072544) and Lipofectamine CRISPRMAX Cas9 Transfection Reagent (1.5 µl of Cas9 Plus and 0.9 µl of CRISPRMAX) (Thermo Fisher, CMAX00008) in a total 100 µl volume of calcium switch media. Organoids were incubated for 2 h and then were centrifuged in the 96‐well plate at 120 rcf and 37°C for 35 min. This extended centrifugation time was used specifically for organoids, which have a more complex structure, as opposed to undifferentiated iPS cells. After centrifugation, 100 µl of Opti‐Mem media (Thermo Fisher, 1985062) was added to each well, then replaced with 200 µl of RB medium 24 h after transfection.

### 4.10 ‘Fluorescence‐On’ Editing of Intact Organoids

*AAVS1* ^LSL‐tdTom^ kidney organoids (day 21), cardiomyocytes (day 21), hepatocytes (day 21), brain and retinal organoids (day 42) or lung organoids (day 28) in 96 well plates were transfected with 110 pmol of Alt‐R S.p. HiFi Cas9 Nuclease V3 (IDT, 1081061), 90 pmol of *AAVS1* ^LSL‐tdTom^ ‐sgRNAs (stoichiometric mixture of *AAVS1* ^LSL‐tdTom^ ‐sgRNA 1, 2, and 3, defined below) and 200 pmol peptide ppTG21^+^ in a total 100 µl volume of calcium switch media. The organoids were incubated for 2 h 30 min and then proceeded to centrifuge at 120 rcf, 37°C, and 35 min. After centrifugation, the organoids were added on 100 µl of Opti‐Mem media (Thermo Fisher, 1985062). 24 h after transfection, the organoids culture was replaced with 200 µl of the appropriate growth media (RB for kidney, RB without insulin for cardiomyocyte, BM for lung).

*AAVS*1^LSL‐tdTom^ ‐sgRNA 1: 5’‐TAGAGGATCTGCGACTCTAG‐3’

*AAVS1* ^LSL‐tdTom^ ‐sgRNA 2: 5’‐ATAAGATACATTGATGAGTT‐3’

*AAVS1* ^LSL‐tdTom^ ‐sgRNA 3: 5’‐GGATCCCCATCAAGCTGATC‐3’

tdTomato positive cells in these cultures were detected 5 days after transfection.

### 4.11 Preparation of MTAs

Alexa Fluor 488 Conjugating kit (Fast)‐Lightning Link (Abcam, ab236553) was used with these two antibodies:

1. Goat anti‐human Podocalyxin antibody (R&D system, AF1658) for PodoTracker
2. Rat anti‐mouse E‐Cadherin antibody [DECMA‐1]‐ Intercellular Junction Antibody (Abcam, ab11512) for TubuleTracker

1 µL of Modifier reagent was added to each 10 µL of antibody to be labeled and mixed gently. The antibody sample (with added Modifier reagent) was pipetted directly onto a vial of lyophilized FITC Conjugation Mix. The mixture was resuspended gently by withdrawing and re‐dispensing the liquid once or twice using a pipette. The mixture was left standing for 15 min in the dark at room temperature (20–25°C). After incubation, 1 µL of Quencher reagent was added for every 10 µL of antibody used, and the mixture was mixed gently. The conjugate could be used after 30 min. The conjugates did not require further purification and were stored at 4°C until use.

### 4.12 Amphiphilic Peptides

Peptides ppTG21 (GLFHALLHLLHSLWHLLLHAC), ppTG21^+^ (Ac‐KLFHALLHLLHSLWHLLLHAC; Ac refers to acetylated N‐terminus), E5TAT (GLFEAIAEFIENGWEGLIEGWYGGRKKRRQRRR), and INF7TAT‐G1K (KLFEAIEGFIENGWEGMIDGWYGYGRKKRRQRR) were generated via custom solid phase synthesis (CPC Scientific Inc; 95% purity). All peptides were stored lyophilized as 10 or 5 mm stocks in 100% DMSO in a desiccator at −20°C.

### 4.13 Generation of a Novel Cas9‐protein G Fusion Protein

An SpCas9 construct featuring three distinct nuclear localization signal (NLS) sequences [58] was augmented near the N terminus (following the first NLS) with a fused 54 amino acid fragment of protein G capable of binding to the constant region of IgG antibodies [59]. This Cas9 construct was expressed in *E. coli* and purified via nickel affinity chromatography (using a detergent wash to remove lipopolysaccharides), heparin affinity chromatography, and size exclusion chromatography as previously described [13]. Purified protein was concentrated to ∼50 µm in a buffer of 20 mm HEPES‐KOH pH 7.5, 150 mm NaCl, 10% (v/v) glycerol, and stored at −80°C.

prG‐triNLS sequence:

SRAAPAAKKKKLDNATYKLILNGKTLKGETTTEAVDAATAEKVFKQYANDNGVDGEWTYDDATKTFTVTEGSAGASDKKYSIGLDIGTNSVGWAVITDEYKVPSKKFKVLGNTDRHSIKKNLIGALLFDSGETAEATRLKRTARRRYTRRKNRISYLQEIFSNEMAKVDDSFFHRLEESFLVEEDKKHERHPIFGNIVDEVAYHEKYPTIYHLRKKLVDSTDKADLRLIYLALAHMIKFRGHFLIEGDLNPDNSDVDKLFIQLVQTYNQLFEENPINASGVDAKAILSARLSKSRRLENLIAQLPGEKKNGLFGNLIALSLGLTPNFKSNFDLAEDAKLQLSKDTYDDDLDNLLAQIGDQYADLFLAAKNLSDAILLSDILRVNTEITKAPLSASMIKRYDEHHQDLTLLKALVRQQLPEKYKEIFFDQSKNGYAGYIDGGASQEEFYKFIKPILEKMDGTEELLVKLNREDLLRKQRTFDNGSIPHQIHLGELHAILRRQEDFYPFLKDNREKIEKILTFRIPYYVGPLARGNSRFAWMTRKSEETITPWNFEEVVDKGASAQSFIERMTNFDKNLPNEKVLPKHSLLYEYFTVYNELTKVKYVTEGMRKPAFLSGEQKKAIVDLLFKTNRKVTVKQLKEDYFKKIECFDSVEISGVEDRFNASLGTYHDLLKIIKDKDFLDNEENEDILEDIVLTLTLFEDREMIEERLKTYAHLFDDKVMKQLKRRRYTGWGRLSRKLINGIRDKQSGKTILDFLKSDGFANRNFMQLIHDDSLTFKEDIQKAQVSGQGDSLHEHIANLAGSPAIKKGILQTVKVVDELVKVMGRHKPENIVIEMARENQTTQKGQKNSRERMKRIEEGIKELGSQILKEHPVENTQLQNEKLYLYYLQNGRDMYVDQELDINRLSDYDVDHIVPQSFLKDDSIDNKVLTRSDKNRGKSDNVPSEEVVKKMKNYWRQLLNAKLITQRKFDNLTKAERGGLSELDKAGFIKRQLVETRQITKHVAQILDSRMNTKYDENDKLIREVKVITLKSKLVSDFRKDFQFYKVREINNYHHAHDAYLNAVVGTALIKKYPKLESEFVYGDYKVYDVRKMIAKSEQEIGKATAKYFFYSNIMNFFKTEITLANGEIRKRPLIETNGETGEIVWDKGRDFATVRKVLSMPQVNIVKKTEVQTGGFSKESILPKRNSDKLIARKKDWDPKKYGGFDSPTVAYSVLVVAKVEKGKSKKLKSVKELLGITIMERSSFEKNPIDFLEAKGYKEVKKDLIIKLPKYSLFELENGRKRMLASAGELQKGNELALPSKYVNFLYLASHYEKLKGSPEDNEQKQLFVEQHKHYLDEIIEQISEFSKRVILADANLDKVLSAYNKHRDKPIREQAENIIHLFTLTNLGAPAAFKYFDTTIDRKRYTSTKEVLDATLIHQSITGLYETRIDLSQLGGDAYPYDVPDYASLGSGSPKKKRKVDGSGSKRPAATKKAGQAKKKKLE.

Residues: 5‐13, PAAKKKKLD, C‐Myc Tag

Residues: 17‐70, YKLILNGKTLKGETTTEAVDAATAEKVFKQYANDNGVDGEWTYDDATKTFTVTE, Protein G

Residues: 1145‐1453, YPYDVPDYA, HA Tag

Residues: 1460‐1466, PKKKRKV, SV40

Residues: 1472‐1487, KRPAATKKAGQAKKKK, Nucleoplasmin

### 4.14 Targeted Editing

10 pmol of MTA was mixed with 180 pmol Protein G‐3xNLS Cas9 and 220 pmol *AAVS1* ^LSL‐tdTom^ ‐sgRNAs in the presence of 200 pmol peptide E5TAT in the total volume of 100 µl of calcium switch media. The organoids were incubated for 2 h 30 min and then proceeded to centrifuge at 120 rcf, 37°C, and 35 min. After centrifugation, the organoids were added on 100 µl of Opti‐Mem media (Thermo Fisher, 1985062). 24 h after transfection, the organoids culture was replaced with 200 ul of the appropriate growth media.

### 4.15 AAV Transduction of Kidney Organoids

Day 18 kidney organoids (AICS36) or Day 28 lung organoids (WTC11) were treated with AAV8‐mCherry (Signagen, SL101128) at a multiplicity of infection (MOI) of 100 000 gc/cell or AAV9‐mCherry (Signagen, SL101129) at a MOI of 100 000 gc/cell, 400 000 gc/cell, and 1 000 000 gc/cell in 100 µl of calcium switch media containing 10 µg/ml polybrene (MiiliporeSigma, TR‐1003‐G) in the calcium shock treatment. Subsequently, organoids were incubated for 2 h 30 min and then proceeded to centrifuge at 120 rcf, 37°C, and 35 min. After centrifugation, the organoids were added on 100 µl of RB media. Without calcium shock treatment, the organoids were only treated with AAV8‐mCherry or AAV9‐mCherry added into 200 µl of RB media (for kidney organoids) or BM media (for lung organoids) containing 10 µg/ml polybrene. Media was again exchanged for fresh RB or BM media 24 h after treatments.

### 4.16 ICE Analysis

Genomic DNA was extracted from transfected cells 48 h post‐transfection using Quick DNA Extraction solution (Lucigen, QE09050) according to the manufacturer's protocol. PCR amplicons across the target site were generated by using GFP locus‐specific primers, forward 5’‐GCAGTGCTTCAGCCGCTAC‐3’ and reverse 5’‐TGAAGTTCACCTTGATGCCGT‐3’. The PCR products were sent for Sanger sequencing (Eurofins). The rate of indel mutations was quantified using ICE analysis (https://ice.synthego.com/#/).

### 4.17 NGS Analysis

Genomic DNA (gDNA) was extracted 48 h post‐delivery using Quick DNA Extraction solution (Lucigen, QE09050) according to the manufacturer's protocol. Concentration of gDNA was assessed either using the NanoDrop spectrophotometer or the Quant‐iT Picogreen dsDNA assay kit (ThermoFisher, P7589) following the manufacturer's instructions.

To generate PCR amplicons across the target site, GFP locus‐specific primers, forward 5’‐GCAGTGCTTCAGCCGCTAC‐3’ and reverse 5’‐TGAAGTTCACCTTGATGCCGT‐3’, were designed using Primer Blast. The amplicon size was 298 bp around the predicted Cas9 cutting site. Amplicon sequencing primers incorporate an adaptor at the 5′ end, making the amplicon compatible with Illumina NGS library preparation.

For PCR, 30–40 ng of gDNA input were used as a template for the high‐fidelity PrimeSTAR GXL DNA Polymerase (Takara, R050B) with the following thermocycler conditions: denaturing for 2 min at 95°C; then 32 cycles of 15 s at 95°C, primer annealing for 15 s at 60°C and amplification of 30 s at 68°C with final amplification of 2 min at 68°C. Amplicons were analyzed by 1% TBE agarose gel electrophoresis, purified by using silica magnetic beads (SPRI) prepared by the UC Berkeley Sequencing Facility, comparable to AMPure XP (Beckman, A63880), and quantified by NanoDrop spectrophotometer (Thermo Scientific, 701‐059049).

Purified PCR products were ligated to Illumina TruSeq adaptors and purified again using SPRI beads. Libraries were prepared at the IGI Sequencing Facility by using a TruSeq DNA Nano HT kit (Illumina, 20015965) according to the manufacturer's guidelines and using a final concentration of 1.36x Sample Purification Beads from the TruSeq kit following end repair for further size selection and followed by high‐throughput sequencing with the MiSeq platform (Illumina, SY‐410‐1003).

Samples were deep sequenced on an Illumina MiSeq at 300 bp paired‐end reads to a depth of ≥ 10 000 reads. As described previously, editing outcomes were assessed using Cortado v1.0 (https://github.com/staciawyman/cortado) [60]. Sequences were adaptor‐trimmed and then joined before performing a global alignment between reads and the reference sequence using NEEDLE [61]. Indel rates were calculated as follows: any reads in which an insertion or deletion overlaps the cut site or occurs within a 10‐base pair window around the cut site, normalized to the total number of aligned reads.

### 4.18 Oxford Nanopore Analysis

Genomic DNA (gDNA) was extracted 48 h post‐delivery using Quick DNA Extraction solution (Lucigen, QE09050) according to the manufacturer's protocol. Concentration of gDNA was assessed using the NanoDrop spectrophotometer (Thermo Scientific, 701‐059049) following the manufacturer's instructions. To generate PCR amplicons across the target site, *PKD2* locus‐specific primers, forward 5’‐TTAAGACTTCTGATACGCGCTGA‐3’ and reverse 5’‐ ACTTCAAATACAACTGTCAGCAACA ‐3’ were designed using Benchling CRISPR Design and Analyze Guides tool with base editing selected as the design type. The amplicon size was 550 bp around the predicted Cas9 cutting site. For PCR, 30–40 ng of gDNA input were used as a template for SuperPlex Premix Polymerase (Takara, 639546) with the following thermocycler conditions: denaturing for 2 min at 95°C; then 35 cycles of 10 s at 95°C, primer annealing for 15 s at 55.5°C, and amplification of 15 s at 72°C. Amplicons were analyzed by 1% TBE agarose gel electrophoresis, purified by using ExoSAP‐IT Express PCR Product Cleanup (Thermo Fisher, 75001.200.UL), and quantified by NanoDrop spectrophotometer (Thermo Scientific, 701‐059049). PCR products were sent for Nanopore sequencing (Eurofins). The rate of mutations was quantified using calculations based on the percentage of C converted to T in total nucleotide at the primary base‐edited site.

### 4.19 Genotoxicity Assay

As a positive control for genotoxicity, day 24 organoids were given a 24‐h pulse with 3 µm mitomycin C (STEMCELL technologies, 73274) and then immediately fixed and stained for gamma H2AX (Cell Signaling, 9718S).

### 4.20 Dextran Accumulation Assay

An 18‐h pulse of 50 µm dextran (Invitrogen, D1820) was administered to live organoids in 96‐well plates in 100 µl of normal cell media (RB). After the pulse, 75 µl of media was removed from the well and replaced with 175 µl of fresh media. This process was then repeated to remove any residual unbound dextran from the well. The organoids were then imaged live using confocal microscopy. Positive control for disrupted physiology was accomplished by treating kidney organoids with a solution of 2 µm latrunculin A (Millipore Sigma, L5163) and RB for 18 h before live cell imaging. For experiments using the live conjugated LTL label (bioWORLD, 21761117‐1), antibodies were administered simultaneously with dextran solution and given the same 18‐h pulse. Immediately before all live cell imaging, sample wells were washed of background dextran using RB media, and identical organoids were reimaged after fixing and staining.

### 4.21 Immunogenicity Assay

Immunogenicity assays were performed by administering a 1 µg IFN‐g (Peprotech, 300‐02) and RB solution to day 23 organoids for 48 h immediately before fixing and staining for APOL1 (4.17A5) (Genentech). Relative mRNA expression of APOL1 and ISG‐15 was measured via qPCR analysis (differences in cycle count to threshold) in organoids given the CRISPR‐RNP complex, g‐IFN, or RB. Well, lysates were collected on day 25 using Trizol (Ambion, 15596026) and processed using TURBO DNase (Invitrogen, AM1907) and Superscript IV first strand synthesis (Invitrogen, 18091050). qPCR primers examined is as follows:

APOL1 Forward: 5’‐GGGAAGATTCCTTGGAGGAGGC‐3’

APOL1 Reverse: 5’‐ATCTGTCCCACTTGGAACGTTTT‐3’

ISG‐15 Forward: 5’‐GAGAGGCAGCGAACTCATCT‐3’

ISG‐15 Reverse: 5’‐CTTCAG CTCTGACACCGACA‐3’

Actin Forward: 5’‐GCGAGAAGATGACCCAGATCAT‐3’

Actin Reverse: 5’‐GGATCTTCATGAGGTAGTCAGTC‐3’

### 4.22 LDL Assay

LDH‐Glo cytotoxicity assay was performed according to manufacturer's protocol (Promega, J2380). Briefly, 25 µL of sample media per well was transferred to a white 96‐well plate (Greiner Bio‐One, 655098). Then, 25 µL of LDH detection enzyme mix and 0.125 µL of reductase substrate were added to each sample well. The plate was incubated at room temperature for 60 min, then luminescence was recorded on a Perkin Elmer Envision 2104 Multilabel reader.

### 4.23 Calcium Imaging With Fluo‐4AM

Undifferentiated *AAVS1* ^LSL‐tdTom^ iPS cells at 3 000 cells/well were plated in 96‐well plates pre‐coated with 60 µL of DMEM‐F12 (Gibco, 11320‐033) containing 0.2 mg/mL GelTrex. 48h after seeding, cells were incubated with 10 µm of Fluo‐4 AM (Thermo Fisher F14201) in 100 µl volume of mTESR1 (STEMCELL Technologies, 85850). Cells were incubated for 20 min to create a baseline for both with and without calcium shock and were then transfected with 10 pmol of Alt‐R S.p. HiFi Cas9 Nuclease V3 (IDT, 1081061), 12 pmol of sgRNA targeting *AAVS1* ^LSL‐tdTom^ ‐sgRNAs (stoichiometric mixture of *AAVS1* ^LSL‐tdTom^ ‐sgRNA 1, 2, and 3, defined below) and Lipofectamine CRISPRMAX Cas9 Transfection Reagent (1.5 µl of Cas9 Plus and 0.9 µl of CRISPRMAX) (Thermo Fisher, CMAX00008) in a total 100 µl volume of either calcium switch media (DMEM (1X) (21068‐028, Gibco) supplemented with 1X GlutaMAX (35050061, Thermo Fisher), 1x Sodium Pyruvate 100 mm (11360‐070, Gibco), 10 ng/ml Recombinant Human FGF‐basic (100‐18B, Peprotech) (with calcium shock method) or Opti‐Mem media (Thermo Fisher, 1985062) (without calcium shock method). Both media were supplemented with 10 µm of Fluo‐4 AM. Cells were centrifuged at 120 rcf and 37°C for 15 min. After centrifugation, 100 µl of mTESR1 supplemented with 10 µm of Fluo‐4 AM was added to each well. Live imaging was performed at 4‐5and 24‐25 h time points.

### 4.24 Image/Video Collection

Live cell imaging of the calcium switch in ZO1‐GFP^+^ iPS cells and organoid experiments, and of *AAVS1^LSL‐tdTom^* cardiomyocytes, was performed on a Yokogawa W1 Spinning Disk confocal head mounted on an inverted Nikon Ti widefield microscope inside of a live cell imaging chamber in a 5% CO_2_ atmosphere at 37°C. Phase contrast and fluorescence images were taken every 24 h. Images of fixed samples were collected on the same microscope.

### 4.25 Immunofluorescence Analysis

Prior to staining, an equal volume of 8% paraformaldehyde (Electron Microscopy Sciences, 15711) was added to the culture media (4% final concentration) for 15 mins at room temperature. After fixing, samples were washed in PBS, incubated with blocking buffer (PBS (Gibco, 10010‐23) + 5% donkey serum (Millipore Sigma, S30‐100ML) + 0.3% Triton‐X‐100 (Sigma Aldrich, X100‐500 mL)), then incubated overnight in antibody dilution buffer comprising PBS + 1% bovine serum albumin + 0.3% Triton‐X‐100 + 10 µm CaCl_2_ (Fisher, AC123350000) with added primary antibodies. Imaging was performed after washing, incubation with Alexa‐Fluor secondary antibodies (Invitrogen), DAPI (ThermoFisher 62248, 1:100) in antibody dilution buffer, and then rinsed for imaging. Primary antibodies and labels included PODXL (R&D system AF1658, 1:400), biotinylated LTL (Vector Labs B‐1325, 1:400), WT1 (Abcam ab89901, 1:500), ECAD (Abcam ab11512, 1:500), gamma H2AX (Cell Signaling 9718S, 1:500), APOL1 (Genentech A.17A5, 1:300), β‐Tubulin III (Sigma Aldrich, T2200), MLC2A (Synaptic System, 311011), CD144 (BD Biosciences 555661, 1:300), MUC1 (Abcam ab15481, 1:200), P63 (R&D BAF1916, 1:40) and NKX2.1 (Seven Hills Bioreagents WRAB‐1231, 1:500). Fluorescence images were captured using a Yokogawa W1 Spinning Disk confocal head mounted on an inverted Nikon Ti widefield microscope with objectives ranging from 20X to 40X.

### 4.26 Image Analysis for AAV9‐mCherry Transduction in Kidney Organoids

Any images that were directly compared to one another were acquired using the same staining and imaging conditions to avoid introducing bias. A 10x confocal z‐stack image of the full organoid was taken, a maximum intensity projection was generated, and then split into individual channels. A threshold was applied to each channel to remove the background signal and highlight the regions of interest. Each channel's threshold values were kept constant across the full set of images being quantified for a given experiment. The thresholded area was then calculated using a particle analyzer, and overlapping regions of interest were calculated using Image Math as detailed in the provided IJ1 Macro. The raw ImageJ script, written as an IJ1 Macro, is available as a supplementary methods section with accompanying notes on necessary user edits for adaptation. Microsoft Excel was used to process the data, and GraphPad Prism was used to visualize the data and perform statistical analyses.

### 4.27 Cell Preparation for Endocytosis in MDCK Cells

Stably transfected EEA1‐GFP positive Madin‐Darby canine kidney (MDCK) cells were kindly provided by Oddmund Bakke's lab. The cells were seeded in two MatTek glass‐bottom imaging dishes (MatTek Corporation, Catalog number P35G‐1.5‐14‐C) and allowed to adhere for 24 h prior to the calcium switch (CaSH) experiment, achieving a final confluency of approximately 50% at the time of analysis. For the CaSh experiment, a calcium‐free medium was prepared by mixing 75% of the total volume of calcium‐free DMEM (high glucose, no glutamine, no calcium) (Thermo Fisher Scientific, Catalog number 21068028) supplemented with 10% fetal bovine serum (FBS) and glutamine with 25% of the total volume of regular culture medium (DMEM containing calcium, supplemented with 10% FBS and glutamine). This preparation was aliquoted and designated as “25% calcium media,” while the regular DMEM containing calcium was aliquoted as “100% calcium media.” To enable visualization, Dextran, Alexa Fluor 647 (10 000 MW, Anionic, Fixable) (Thermo Fisher Scientific, Catalog number D22914) was dissolved in both media to achieve a final concentration of 10 µg/ml. The dextran‐containing media were then added to the two imaging dishes containing the MDCK cells, which were incubated in a 5% CO_2_ atmosphere for 30 min. Following incubation, the cells were fixed using 4% paraformaldehyde (PFA) for 15 min at room temperature. Imaging of the fixed cells was performed using both a Nikon Ji Microscope equipped with a Crest Spinning Disk (60X/1.27 NA) and an MI‐SIM Microscope from CSR operating in TIRF‐SIM mode (60X/1.42 NA). Z‐stacks were acquired for further analysis. Colocalization of the dextran channel and the EEA1 channel was analyzed using ImageJ2 (Fiji) with the M1 and M2 coefficients obtained from the JaCoP plugin. Prior to colocalization analysis, images were denoised using NIS Denoise.ai (Nikon) to enhance the signal‐to‐noise ratio. Background subtraction was performed using the rolling ball algorithm, and thresholds were automatically determined via the ImageJ AutoThreshold function. Each experiment was repeated twice, yielding a total of 10 images for analysis. Each field of view contained approximately 5 to 10 cells, resulting in a final sample size of around 50 cells. The M1 and M2 coefficients, representing the fraction of overlap between the two channels, were computed for each field of view to assess the extent of colocalization.

### 4.28 Quantification and Statistical Analysis

#### 4.28.1 Statistical Analysis

Quantification was performed on data obtained from experiments performed on controls and treatment conditions side by side on at least three different occasions or cell lines (biological experiments). Error bars are mean ± standard error (s.e.m.). Statistical analyses were performed using GraphPad Prism Software. To test significance, *p*‐values were calculated using a two‐tailed, unpaired t‐test with Welch's correction (unequal variances) or ANOVA (with multiple comparisons). Statistical significance was defined as *p* < 0.05. Exact *p*‐values are provided in the figure legends in experiments that showed statistical significance.

## Author Contributions

B.S.F. and R.W. conceived the project with input from N.V. N.V., B.S.F., R.W., L.O., X.H., D.H., H.F., and R.J.M. designed the experiments. N.V., L.O., M.F., X.H., S.B., D.V.F., J.V., and K.A. performed the experiments and analyzed the data. N.V., L.O., R.W., and B.S.F. wrote the paper with input from all authors.

## Conflicts of Interest

B.S.F. is an inventor on patents including “Three‐dimensional differentiation of epiblast spheroids into kidney tubular organoids modeling human microphysiology, toxicology, and morphogenesis” and “High‐throughput automation of organoids for identifying therapeutic strategies”. B.S.F. holds an ownership interest in Plurexa LLC. R.C.W. is a co‐founder, shareholder, and compensated consultant of Editpep, Inc. None of the preceding interests affected in any way the results of the paper.

## Supporting information




**Supporting File 1**: advs73126‐sup‐0001‐SuppMat.pdf.

**Supporting File 2**: advs73126‐sup‐0002‐Movie1‐b.m4v.

## Data Availability

All datasets, including raw data and statistical analysis, are available upon reasonable request from the corresponding author. Source data is publicly available through the SCGE Toolkit. iPS cell lines used in this study may be obtained from the corresponding authors upon request and in accordance with material transfer agreements from the University of Washington, University of California, and any third‐party originating sources.
