# Supplementary material for: Calcium Shock Enables Efficient and Programmable Particle Delivery for Genome Editing Applications
Source: Adv Sci (Weinh). 2026 Mar 17;13(21):e10441. doi: 10.1002/advs.202510441 (PMC13073298; doi:10.1002/advs.202510441)
Supplement: Supplementary file 1 — Supporting File 1: advs73126‐sup‐0001‐SuppMat.pdf. [file ADVS-13-e10441-s001.pdf]

Figure S1. Calcium shock increases transfection and genome editing in iPS cells, related to Figure 1.

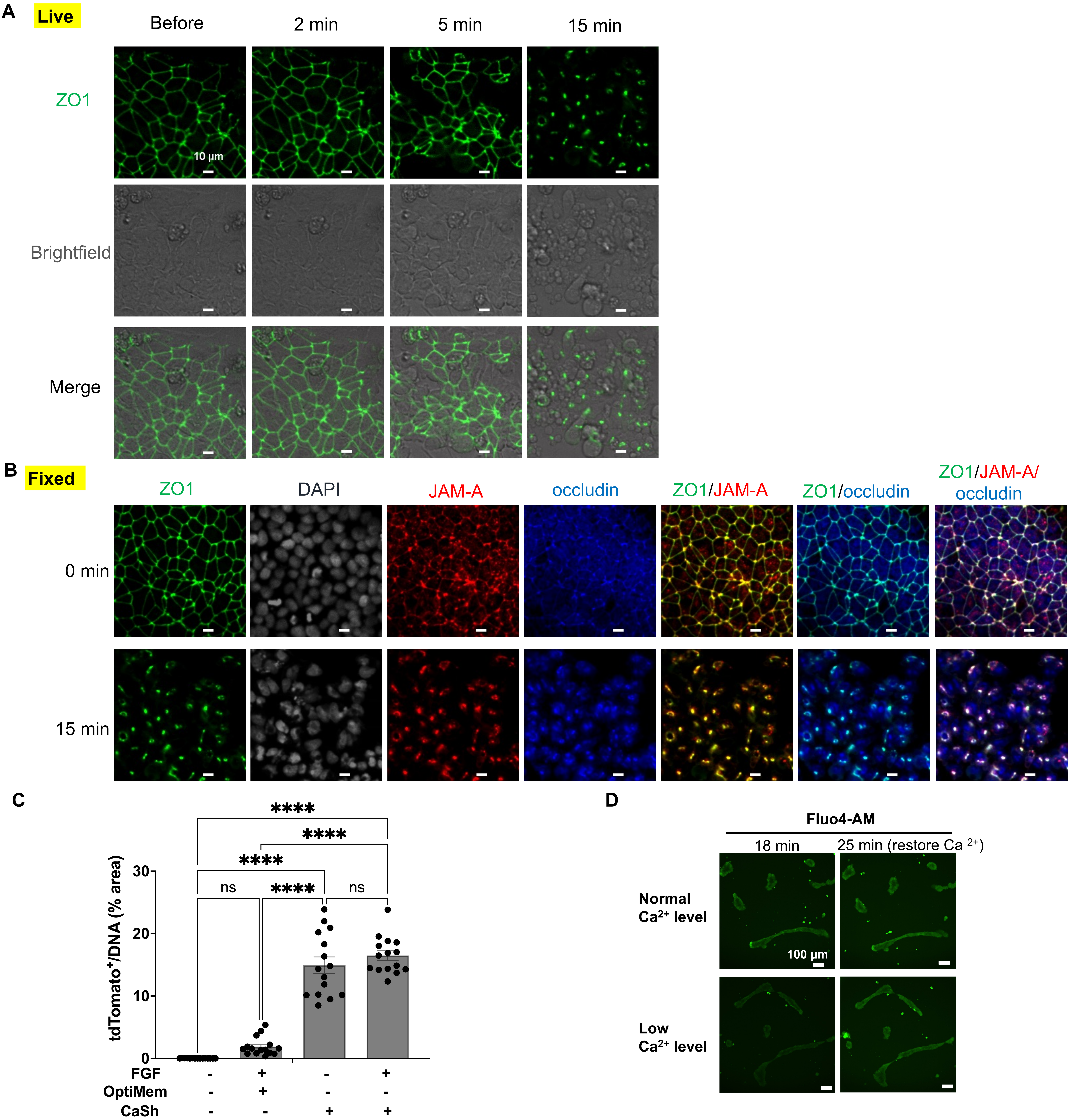

Figure S1 (continued). Calcium shock increases transfection and genome editing in iPS cells, related to Figure 1

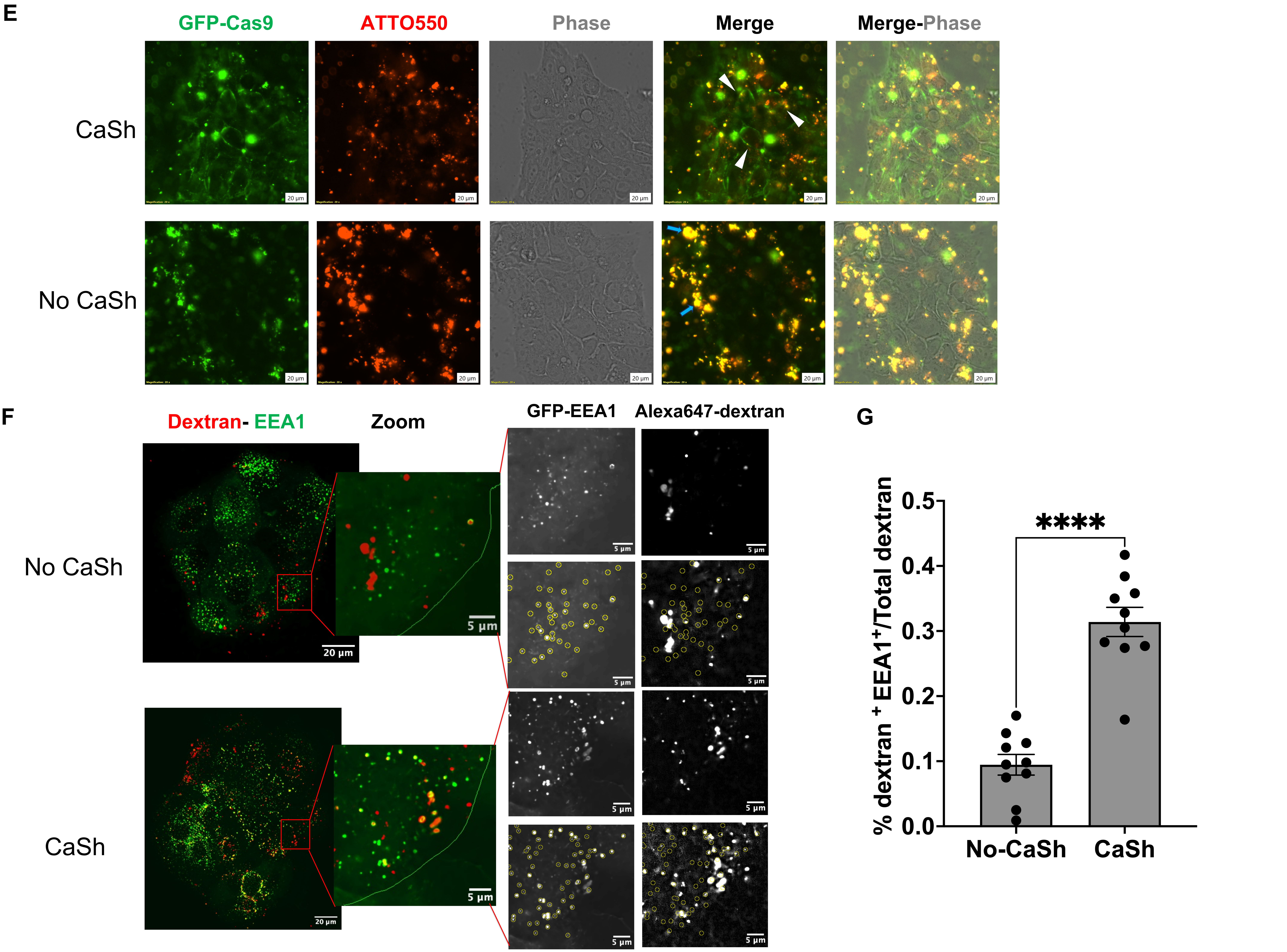

Figure S1. Calcium shock increases transfection and genome editing in iPS cells, related to Figure 1.
(A) Time course showing live confocal images of a representative colony of iPS cells expressing ZO1-GFP after being switched into CaSh media, or **(B)** representative immunofluorescence images of fixed iPS cell colonies under this treatment condition. The gene name for occludin is *OCLN.* **(C)** Quantification of confocal immunofluorescence images of fluorescence-on iPS cell subjected to genome editing conditions with or without FGF treatment (mean ± s.e.m., n = 15 images per condition, pooled from three independent biological experiments, *ns,* not significant, ****, $p < 0.0001$, Ordinary one-way ANOVA with multiple comparisons) **(D)** Representative confocal images of iPS cell pulsed with Fluo-4 AM in normal or low calcium. **(E)** Representative confocal images of iPS cells transfected with Cas9 RNP complexed with ATTO 550 using CRISPRMAX with and without calcium shock. Arrowheads indicate examples of more specific and intracellular localization patterns. Blue arrows indicate non-specific aggregations outside of cells. **(F)** Representative confocal optical sections and **(G)** quantification of GFP-EEA1 MDCK cells treated with AlexaFluor647-dextran (n=10 images per condition, pooled from two independent biological experiments, ****, $p < 0.0001$, Mann-Whitney test). Yellow circles indicate EEA1$^+$ endosomes.

Figure S2. Calcium shock greatly increases genome editing in multicellular colonies of iPS cells, related to Figure 2.

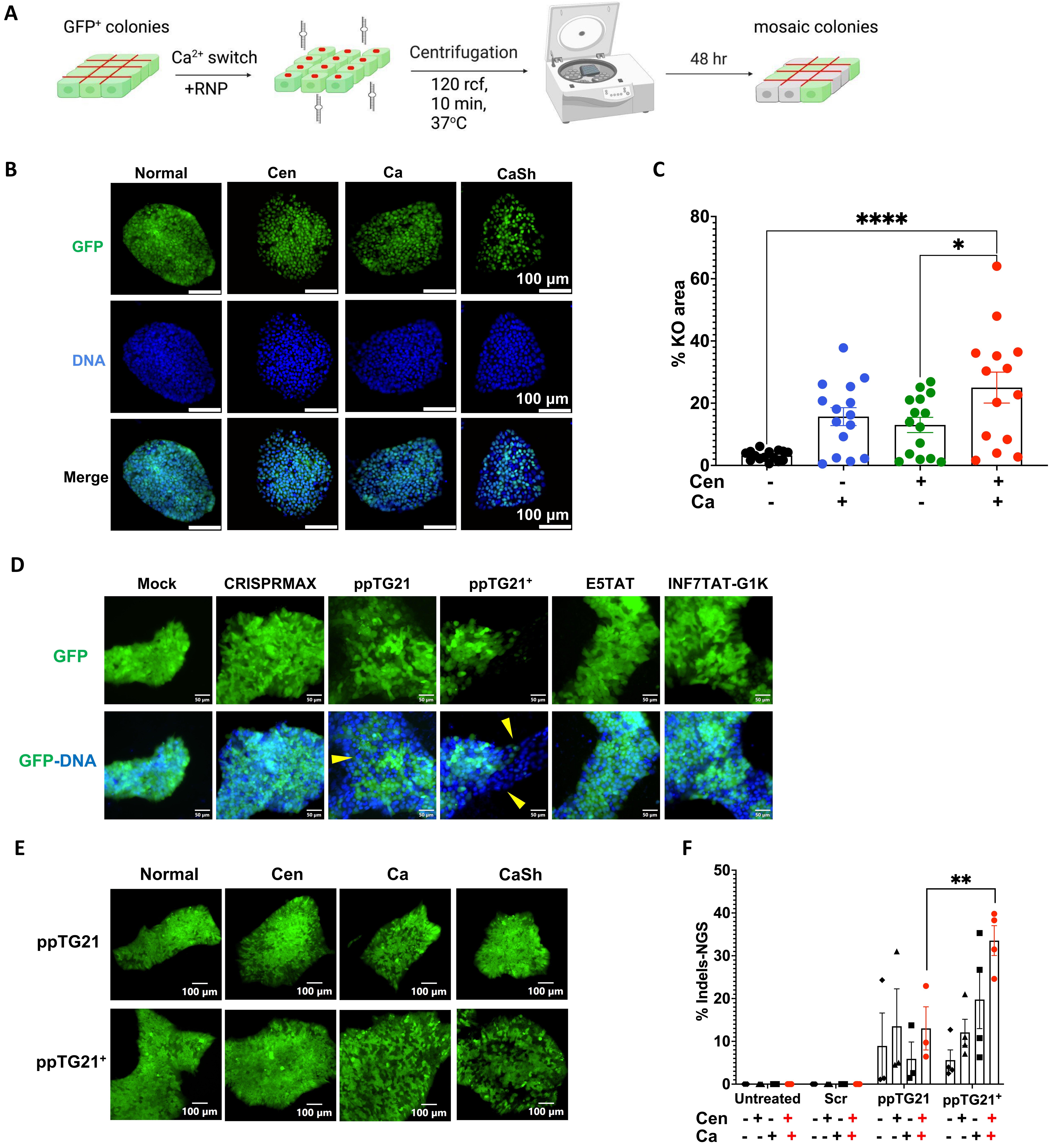

Figure S2. Calcium shock increases genome editing in multicellular colonies of iPS cells, related to Figure 2.
(A) Schematic of calcium switch and centrifugation experiments in colonies. **(B)** Representative confocal images of iPS cell colonies treated with RNP Cas9 gRNA with CRISPRMAX in different conditions: normal, centrifugation, calcium switch, and combined centrifugation/calcium switch (Calcium shock). **(C)** Quantification of the percentage of knock out cells (GFP negative) in (B) (mean ± s.e.m., n = 3 independent biological experiments; *, $p < 0.05$; ****, $p < 0.0001$, Ordinary one-way ANOVA with multiple comparisons). **(D)** Representative confocal images of GFP-expressing iPS single cells treated with RNP Cas9 gRNA targeting a scrambled sequence (Mock) or GFP with different transfectants (CRISPRMAX or peptides ppTG21, ppTG21^{+}, E5TAT, INF7TAT-G1K). CRISPRMAX was used as the transfection reagent for the 'Mock' condition. Arrowheads indicate areas of GFP loss. **(E)** Representative confocal images of iPS cell colonies expressing constitutive GFP, subjected to genome editing treatment with peptide ppTG21 and peptide ppTG21^{+} in different conditions: normal, centrifugation, calcium switch, and Calcium shock. **(F)** Quantification of indel formation based on NGS in GFP-expressing colonies (mean ± s.e.m., n=4 independent biological experiments, **, $p < 0.01$, two-way ANOVA with multiple comparisons).

Figure S3. Calcium shock increases genome editing in organoids, related to Figure 2.

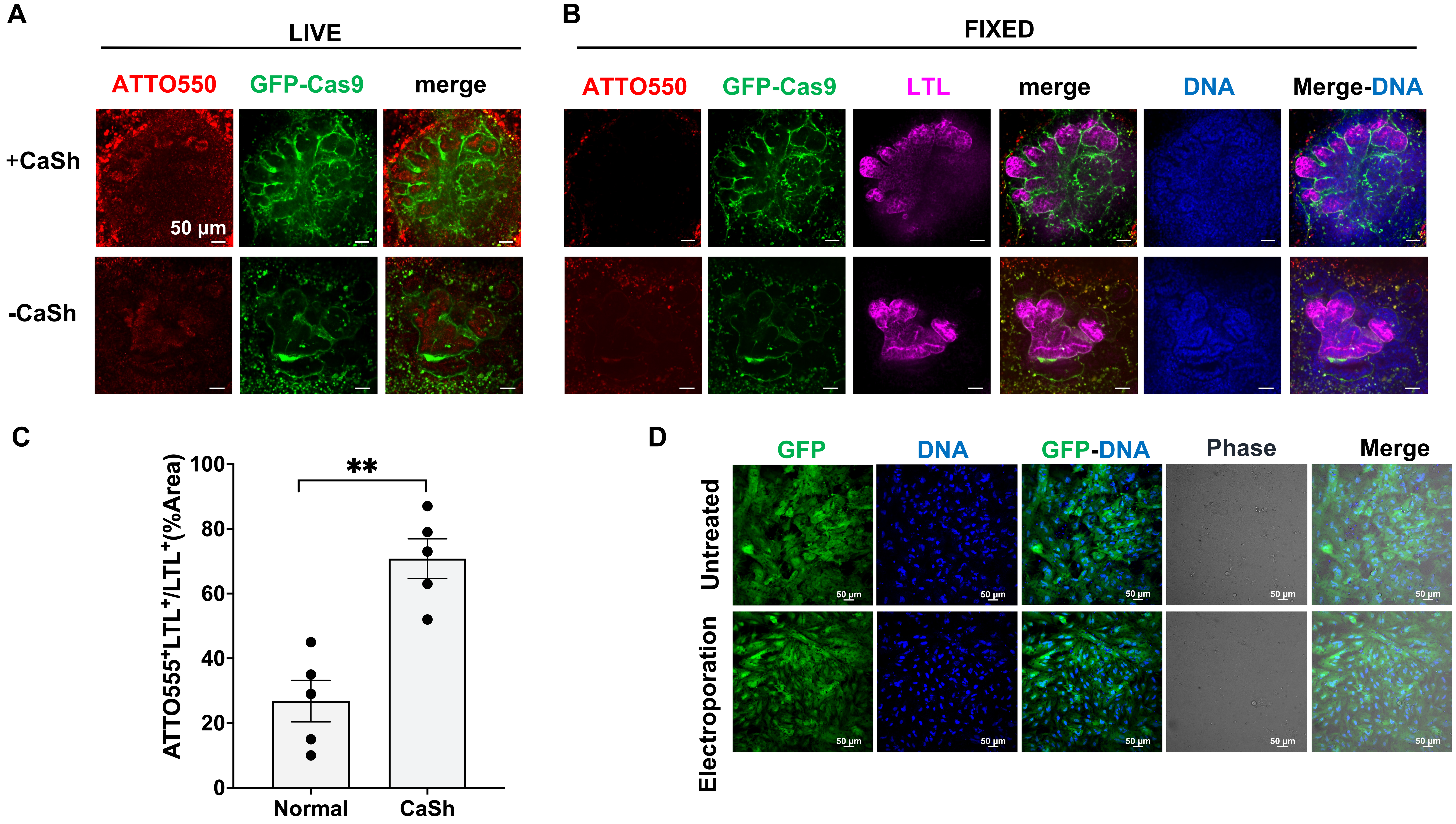

Figure S3. Calcium shock increases genome editing in organoids, related to Figure 2.

(A) Representative confocal images of live and **(B)** fixed organoids transfected with Cas9 RNP complexed with ATTO 550 using CRISPRMAX and Calcium shock. **(C)** Quantification of ATTO 550 in proximal tubules expressing LTL (mean ± s.e.m, n = 3 independent biological experiments per condition, **, $p < 0.01$, Mann-Whitney test). **(D)** Confocal immunofluorescence of GFP-expressing organoid outgrowths ± electroporation with Cas9 RNP. Organoid were replated immediately after electroporation and imaged after 48 hours.

Figure S4. CaSh-Pro enables programmable editing of specific cell types in human organoids, related to Figure 3.

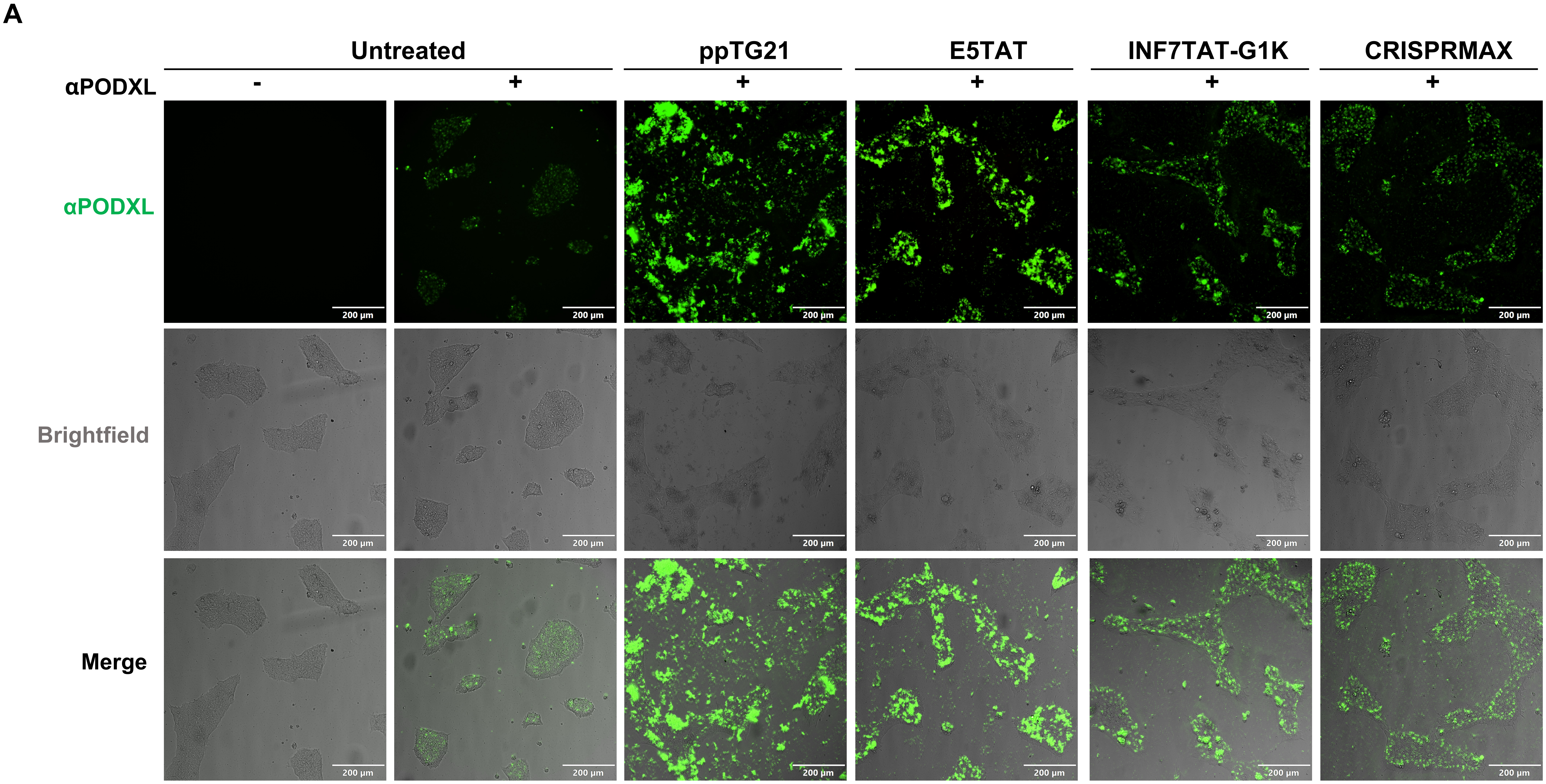

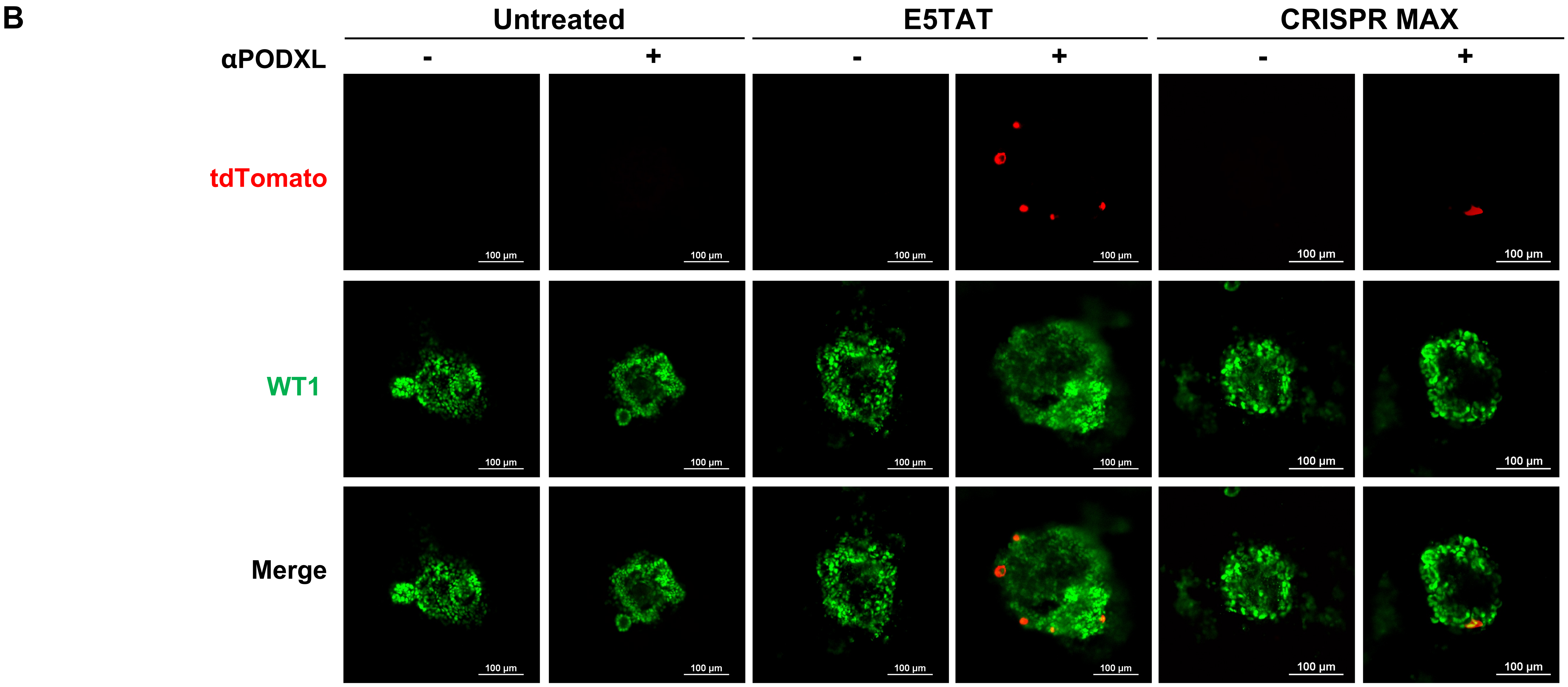

Figure S4. CaSh-Pro enables programmable editing of specific cell types in human organoids, related to Figure 3.

(A) Representative confocal immunofluorescence images of $AAVS1^{LSL\text{-}tdTom}$ iPS cells treated with RNP Cas9 gRNA ± PodoTracker (αPODXL) using different transfection reagents: peptide ppTG21, E5TAT, INF7TAT-G1K, and CRISPRMAX. **(B)** Representative confocal images of $AAVS1^{LSL\text{-}tdTom}$ kidney organoids 5 days after treatment with RNP Cas9 gRNA ± PodoTracker (αPODXL) using different transfection reagents: peptide E5TAT and CRISPRMAX).

Figure S5. Calcium shock enables fluorescence-on genome editing in diverse human organ lineages, related to Figure 4.

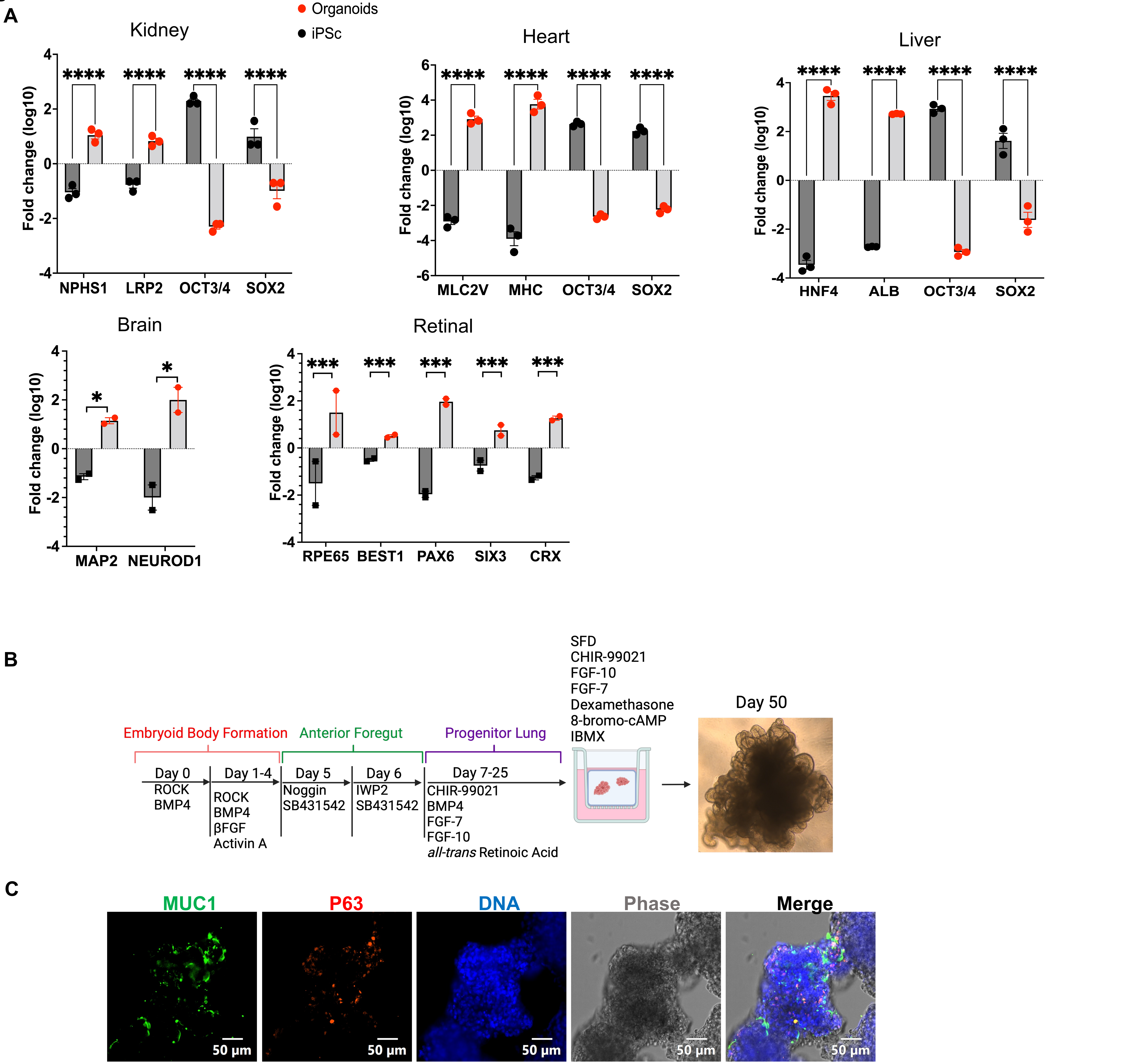

Figure S5 (continued). Calcium shock enables fluorescence-on genome editing in diverse human organ lineages, related to Figure 4.

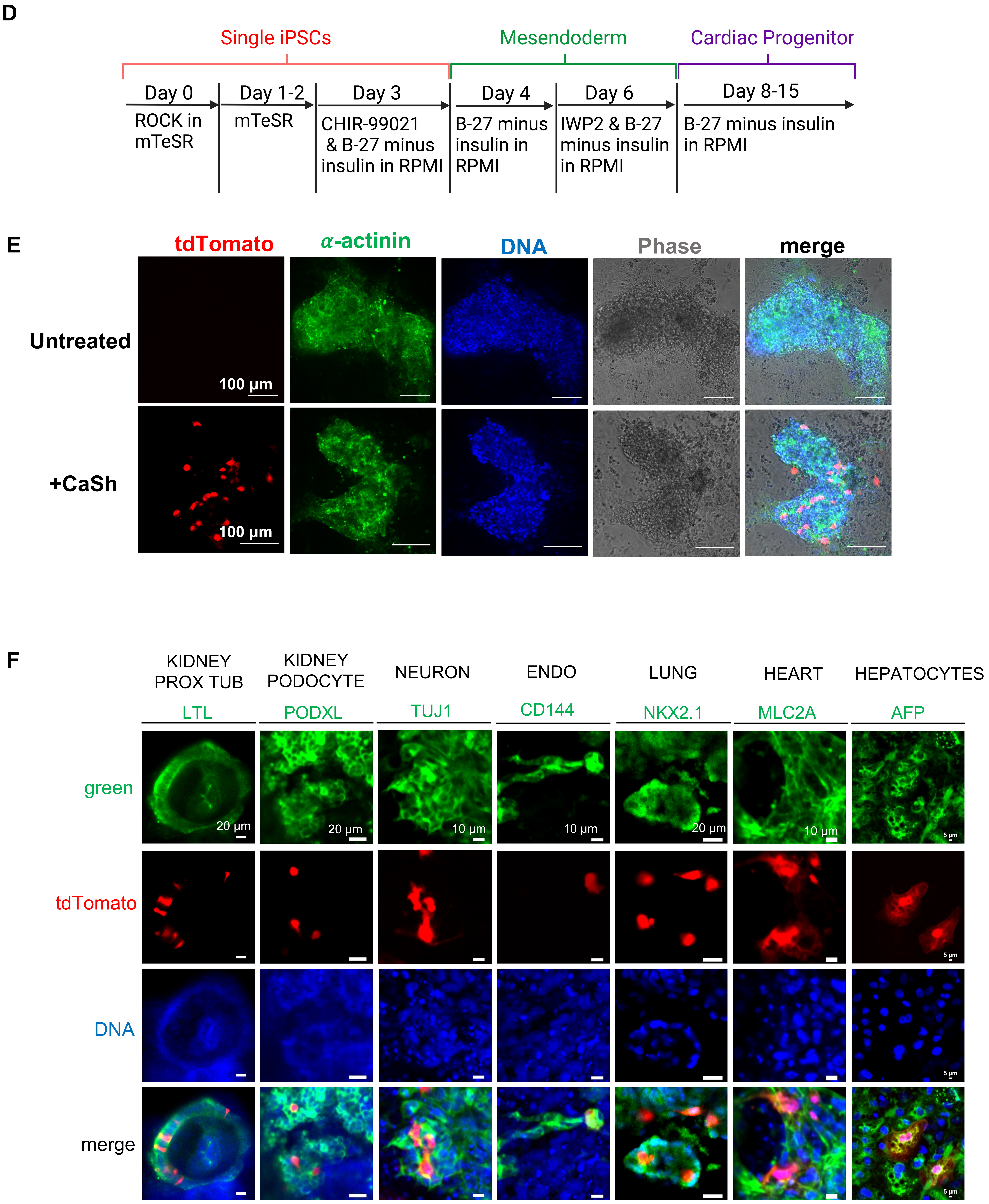

Figure S5: Calcium shock enables fluorescence-on genome editing in diverse human organ lineages, related to Figure 4.

(A) qPCR analysis of marker gene sets associated with five different somatic lineages (kidney, liver, heart, brain and eye), compared to undifferentiated iPS cells as a negative control (mean ± s.e.m, n = 3 independent biological experiments, *, $p < 0.05$, ***, $p < 0.001$, ****, $p < 0.0001$, two-way ANOVA for kidney, heart, liver, unpaired t test for brain and retinal). **(B)** Schematic of lung organoid differentiation, with phase contrast image of final product. **(C)** Confocal immunofluorescence image of lung organoids displaying markers of basal cells (P63) and epithelial mucus cells (MUC1) at day 25. **(D)** Schematic of cardiomyocyte differentiation. **(E)** Confocal immunofluorescence optical sections showing tdTomato reporter expression in cardiomyocytes subjected to genome editing using transfection of Cas9 RNP with CRISPRMAX + CaSh, compared to untreated control. **(F)** Confocal immunofluorescence optical sections showing tdTomato reporter expression in different organ lineages after editing. Kidney, neuron, endothelium, hepatocytes and lung cells were transfected with peptide $ppTG21^{+}$ and cardiomyocytes were transfected with CRISPRMAX.

Figure S6. Organoids recover from calcium shock without detectable adverse events, related to Figure 4.

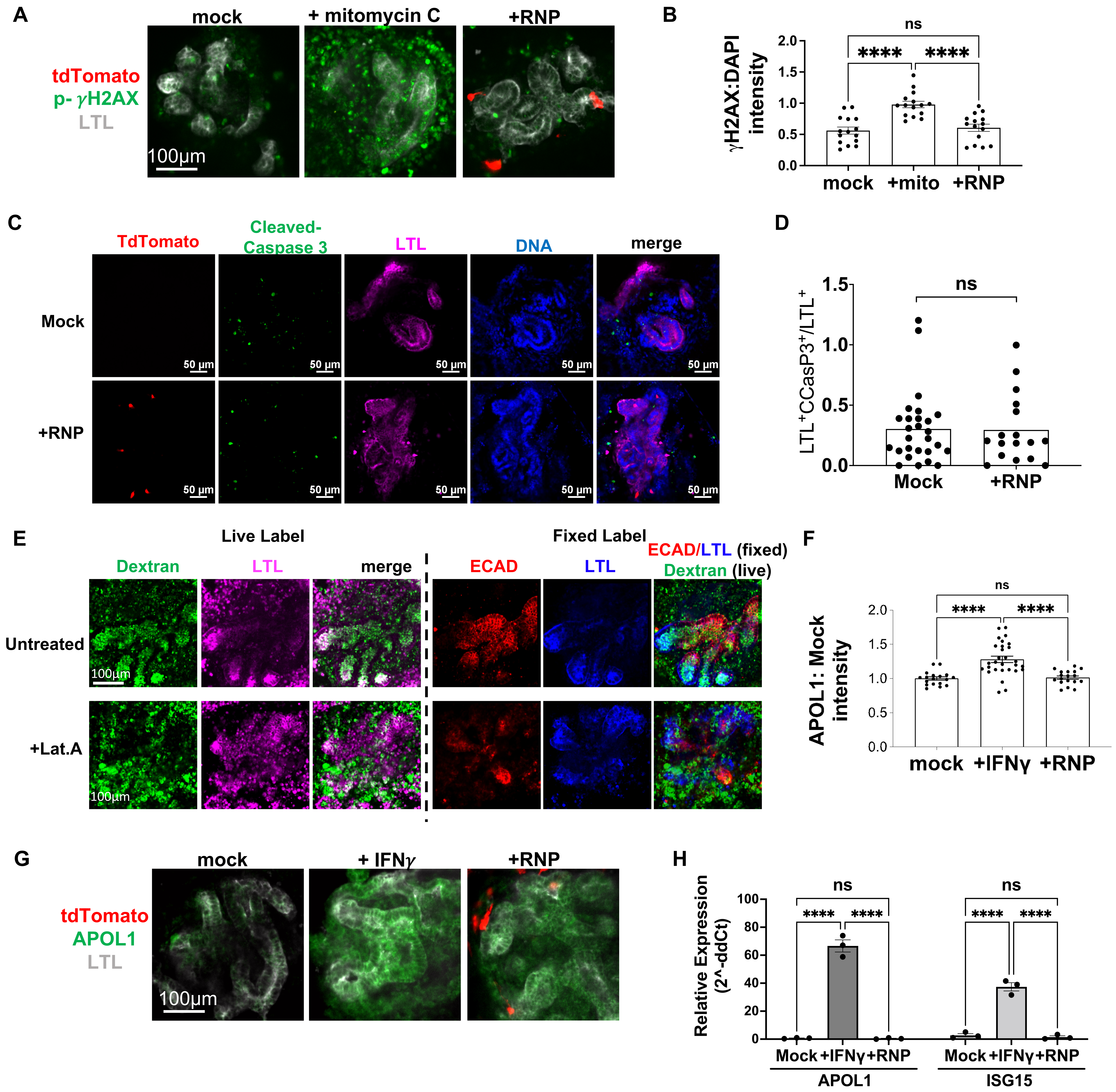

Figure S6 (continued). Organoids recover from calcium shock without detectable adverse events, related to Figure 4.

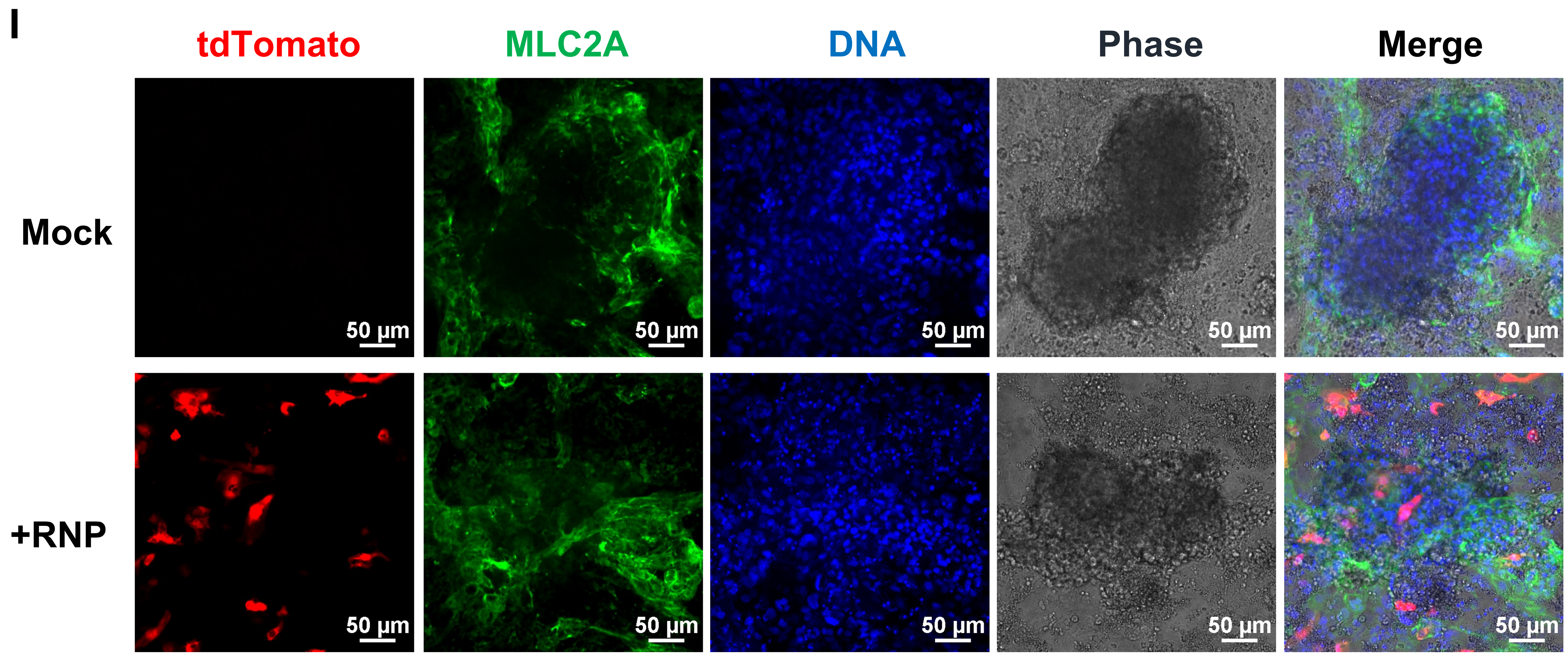

Figure S6. Organoids recover from calcium shock without detectable adverse events, related to Figure 4.
(A) Representative confocal immunofluorescence images and **(B)** quantification of DNA damage (p-γH2AX normalized to DNA) in organoids (mean ± s.e.m, n = 15 organoids per condition pooled from n = 3 independent experiments, *ns*, not significant, ****, $p < 0.0001$, Ordinary one-way ANOVA with multiple comparisons). **(C)** Representative confocal immunofluorescence images of untreated and gene edited organoids stained with cleaved caspase 3, LTL and DAPI with **(D)** quantification of apoptotic cells (mean ± s.e.m, n = 17-27 organoids per condition pooled from n = 3 independent experiments, *ns*, not significant, Mann-Whitney test). **(E)** Representative confocal immunofluorescence images of kidney organoids treated ± latrunculin A, stained with live dextran and LTL-Cy5 labels then reimaged post fixation and stained with ECAD and biotinylated-LTL **(F)** Quantifications of APOL1 immunofluorescence intensity within organoids (mean ± s.e.m., mock and +RNP n ≥ 18 organoids per condition pooled from three independent experiments; *ns,* not significant, ****, $p < 0.0001$, Ordinary one-way ANOVA with multiple comparisons). **(G)** Representative confocal immunofluorescence images of APOL1 expression. **(H)** Relative qPCR analysis of APOL1 and ISG15 (mean ± s.e.m from n = 3 independent biological experiments; each dot represents a biological replicate that was averaged between three technical experiments; *ns,* not significant, ****, $p < 0.0001$, two-way ANOVA with multiple comparisons). **(I)** Confocal immunofluorescence images of cardiomyocyte clusters shown in Movie 1 after fixation and staining.

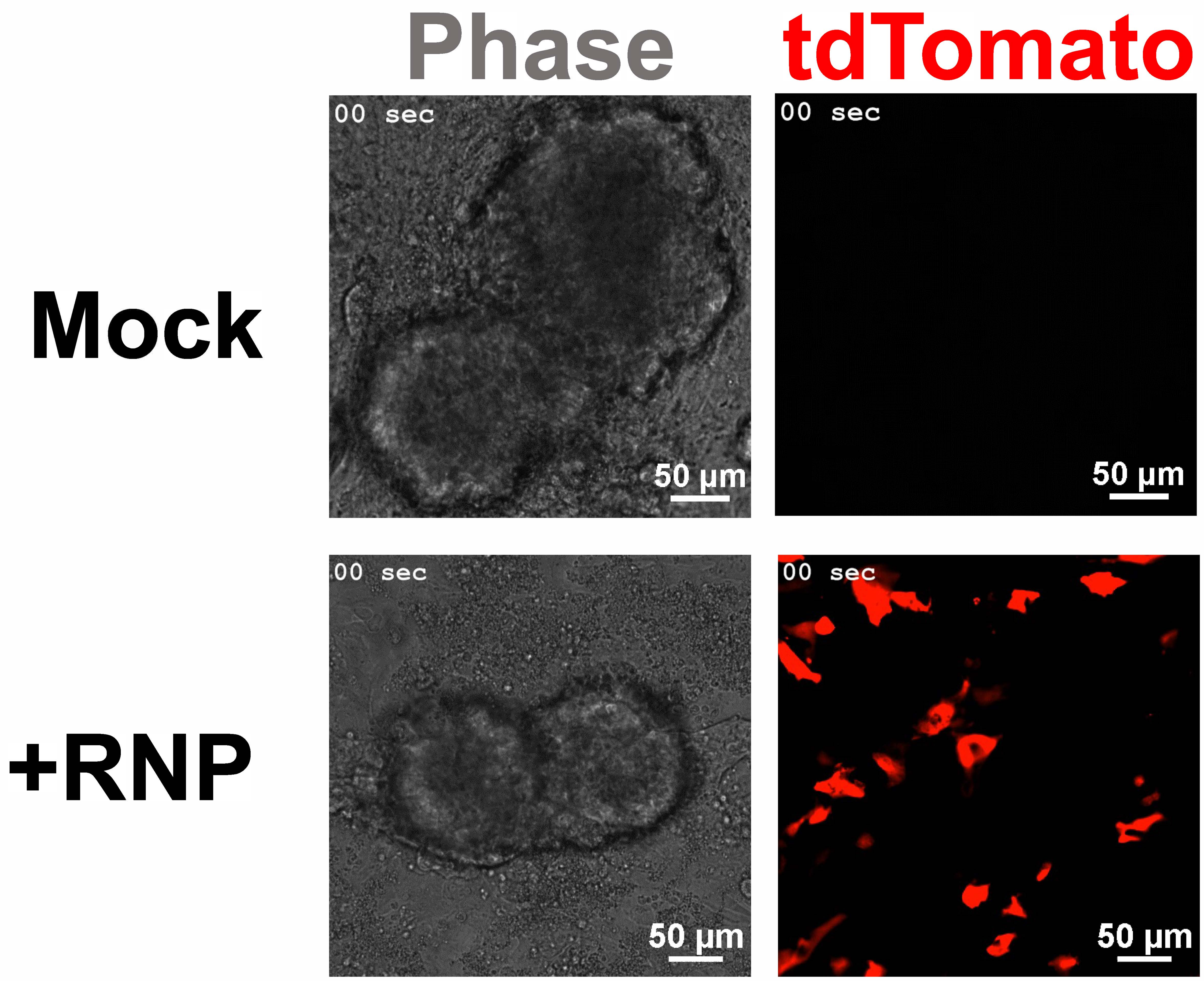

Movie 1. Videos of synchronously beating cardiomyoctes derived from *AAVS1*$^{LSL\text{-}tdTom}$ iPS cells. Untreated and Cas9-RNP treated cardiomyocytes were imaged at day 28 (7 days post transfection). Brightfield video and red fluorescent video were taken separately from each group. Brightfield video was captured at 1 image every 100ms. Red fluorescent video was captured at 1 image every 200ms.
